# ﻿Smooth post-labial chaetae in *Homidia* (Collembola, Entomobryidae) and the description of four new species from China with the aid of DNA barcoding

**DOI:** 10.3897/zookeys.1213.123839

**Published:** 2024-09-25

**Authors:** Rong Zhou, Ling Huang, Yi-Tong Ma

**Affiliations:** 1 School of Life Sciences, Nantong University, Nantong, Jiangsu 226000, China Nantong University Nantong China

**Keywords:** Chaetotaxy, COI, DNA sequences, Guangxi, springtails, taxonomy

## Abstract

Four new species of *Homidia* are described from the Guangxi Zhuang Autonomous Region, China. *Homidialongiantenna***sp. nov.** is characterised by its long antenna and slightly expanded post-labial chaetae; *H.guangxiensis***sp. nov.** by the presence of smooth chaetae on the post-labium and posterior face of the ventral tube; *H.huapingensis***sp. nov.** by the presence of smooth post-labial chaetae and pointed tenent hairs; and *H.oligoseta***sp. nov.** by the pointed tenent hairs and fewer macrochaetae on Abdomen IV. Additions to the original description of *Homidiaacutus* Jing & Ma, 2022 are also provided.

## ﻿Introduction

To date, 77 species of the genus *Homidia* have been described worldwide (Bellinger et al. 1996–2024). The main characters in the taxonomy of the genus include colour pattern, body chaetotaxy, chaetae of the labial base, claw structure and dental spines. The post-labial chaetae are rarely mentioned in species descriptions because they are usually not different from the normal ciliate chaetae present in most species, except for a few expanded ones. Prior to this study, smooth post-labial chaetae had not been reported in the genus. Here, we describe four new species of *Homidia*, among which one species has slightly expanded post-labial chaetae, two have smooth post-labial chaetae and the other one lacks expanded or smooth post-labial chaetae. Additions to the original description of *Homidiaacutus* Jing & Ma, 2022 are also provided.

## ﻿Material and methods

### ﻿Taxon sampling and specimen examination

Specimens were collected with an aspirator and stored in 99% alcohol. They were mounted on glass slides in Marc André II solution and were studied with a Leica DM2500 phase contrast microscope. Photographs were taken using a Leica DFC300 FX digital camera mounted on the microscope and enhanced with PHOTOSHOP CS2 (Adobe Inc.). Type specimens are deposited in the School of Life Sciences Nantong University, Jiangsu, China.

The nomenclature of the dorsal macrochaetotaxy of the head and interocular chaetae follows Jordana and Baquero (2005) and Mari-Mutt (1986). Labial chaetae are designated following Gisin (1964). Post-labial chaetae follow Chen and Christiansen (1993). Labral and tergal chaetae of the body follow Szeptycki (1973, 1979).

### ﻿Molecular analysis

DNA was extracted by using an Ezup Column Animal Genomic DNA Purification Kit (Sangon Biotech, Shanghai, China) following the manufacturer’s standard protocols. Amplification of a 658 bp fragment of the mitochondrial COI gene was carried out using a Prime Thermal Cycler (TECHNE, Bibby Scientific Limited, Stone, Staffordshire, UK), performed in 25 μl volumes using Premix Taq polymerase system (Takara Bio, Otsu, Shiga, Japan). The primers and polymerase chain reaction (PCR) programs followed Greenslade et al. (2011). All PCR products were checked using a 1% agarose gel electrophoresis. Successful products were purified and sequenced on an ABI 3730XL DNA Analyser (Applied Biosystem, Foster City, CA, USA). All procedures were completed by Pucheng (Nanjing, China).

DNA sequences were assembled using SEQUENCHER 4.5 (Gene Codes Corp) and then deposited in GenBank (Table 1). Sequences were aligned using ClustalW implemented in MEGA 5.1 (Tamura et al. 2011) with default settings. Pairwise genetic distances were analysed in MEGA 5.1 employing the Kimura 2-parameter (K2P) model (Kimura 1980).

**Table 1.** Number of individuals, GenBank accession numbers and source of sequences of the species in this study.

Species	Number of individuals	GenBank accession number	Source
*Homidiahuapingensis* sp. nov.	C7401	PP379450	This study
	C7402	PP379451	
	C8201	PP379452	
	C8203	PP379453	
	C8204	PP379454	
	C8202	PP379455	
	C8303	PP379456	
*Homidialongiantenna* sp. nov.	C8107	PP379457	This study
	C8103	PP379458	
	C8104	PP379459	
	C8105	PP379460	
	C8106	PP379461	
*Homidiaguangxiensis* sp. nov.	C8302	PP379462	This study
	C8304	PP379463	
	C8305	PP379464	
*Homidiaoligoseta* sp. nov.	C8306	PP379465	This study
	C8307	PP379466	
	C8308	PP379467	
	C8309	PP379468	
*Homidiaacutus* Jing & Ma, 2022	C44-3-a	PP379469	This study
	C29-2-a	PP379470	
	C29-1-a	PP379471	
	C2902	PP379472	
	C4404	PP379473	

### ﻿Abbreviations

**Ant.** antennal segment(s);

**Th.** thoracic segment(s);

**Abd.** abdominal segment(s);

**mac** macrochaeta(e);

**mes** mesochaeta(e);

**ms** specialised microchaeta(e);

**sens** specialised ordinary chaeta(e).

## ﻿Results

### ﻿Class Collembola Lubbock, 1873

**Order Entomobryomorpha Börner, 1913**

**Family Entomobryidae Tömösvary, 1882**

#### ﻿Genus Homidia

Taxon classificationAnimaliaCollembolaEntomobryidae

Börner, 1906

1516F3EF-4CB1-5C79-B322-155FFCE4A9AF

##### Diagnosis.

Moderate size, usually 1–2 mm; eyes 8+8; antennae four segmented; mucro bidentate and with a basal spine, subapical tooth much larger than apical one; dentes with spines; scales absent on body; macrochaetae on anterior part of Abd. IV arranged as an irregular “collar”.

#### ﻿ Homidia longiantenna sp. nov.

Taxon classificationAnimaliaCollembolaEntomobryidae

98B6E4A3-384A-5F33-9A05-C11BD8873059

https://zoobank.org/67A3996A-CCA2-42CC-BD48-3C878435D11F

Figs 1 , 2−8 , 9−11 , 12−17 , 18 , 19 , 20–27 , Tables 2 , 3

##### Type material.

***Holotype*** • ♀ on slide, China, Guangxi Zhuang Autonomous Region, Guilin City, Longsheng Autonomous County, Huaping Natural Reserve, Tianping Mountain, 31-V-2023, 25°37′52″N, 109°54′47″E, 935.4 m asl, sample number 1281. ***Paratype*** • ♀ on slide, same data as holotype. All collected by Y-T Ma.

##### Description.

***Size*.** Body length up to 3.02 mm.

**Figure 1.**
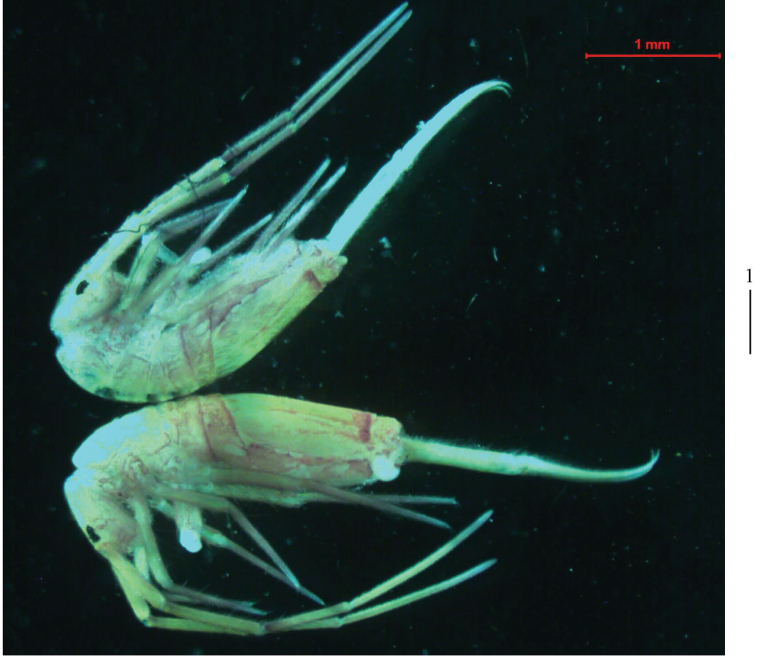
Habitus of *Homidialongiantenna* sp. nov. (lateral view). Scale bar: 500 μm.

***Coloration*.** Ground colour pale yellow; eye patches dark blue; scattered brown pigment present on body, especially tibiotarsus, lateral and posterior parts of Abd. IV, sometimes Ant. III–IV and Abd. II–III also with brown pigment (Fig. 1).

**Figures 2–8.**
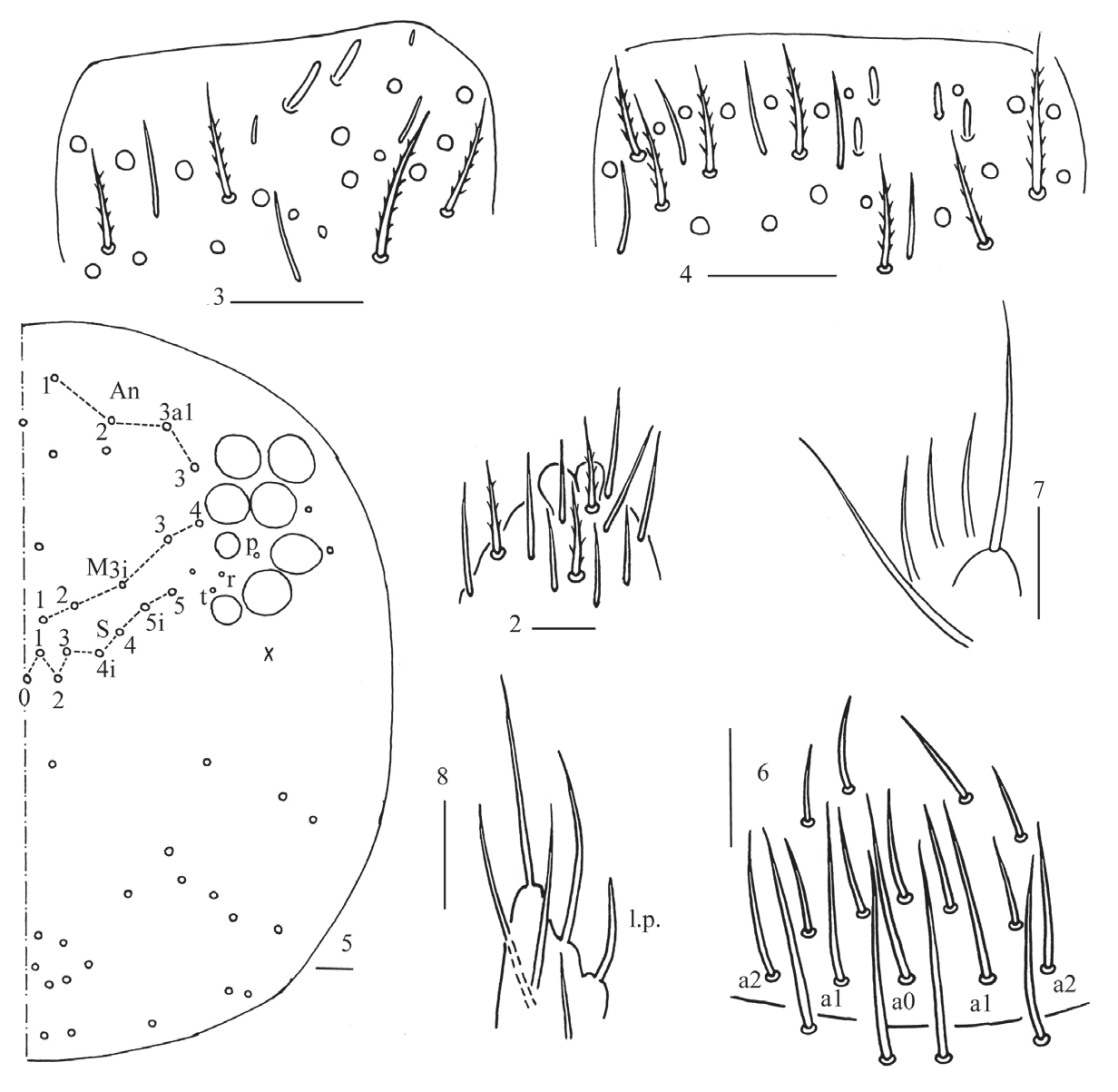
*Homidialongiantenna* sp. nov. **2** apex of Ant. IV (dorsal view) **3** distal Ant. III (ventral view) **4** distal Ant. II (ventral view) **5** dorsal head (right side) **6** prelabrum and labrum **7** maxillary palp and outer lobe (right side) **8** labial palp. Scale bars: 20 μm.

**Figures 9–11.**
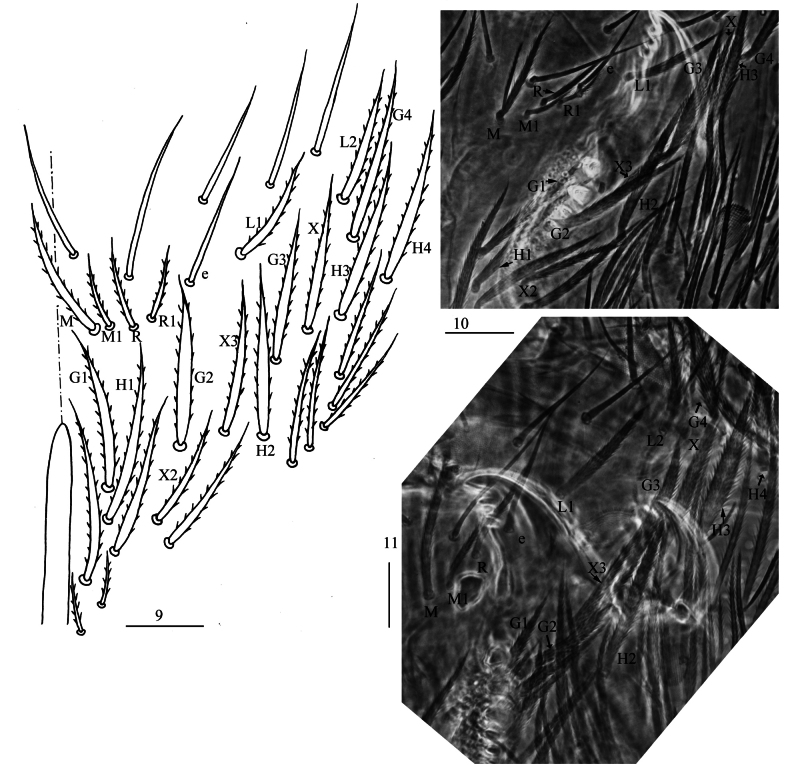
*Homidialongiantenna* sp. nov. **9** labial and post-labial chaetotaxy (right side) **10, 11** photographs of labial and post-labial chaetotaxy (right side). Scale bars: 20 μm.

***Head*.** Antenna not annulated and 0.98–1.07 times length of body. Ratio of Ant. I–IV as 1.00/1.24–1.50/1.06–1.30/2.06–2.60. Distal part of Ant. IV with many sensory chaetae and normal ciliate chaetae, apical bulb bilobed (Fig. 2). Sensory organ of Ant. III with two rod-like chaetae (Fig. 3). Sensory organ of Ant. II with 3(4) rod-like chaetae (Fig. 4). Eyes 8+8, G and H smaller than others, interocular chaetae as p, r, t mes. Dorsal chaetotaxy of head with four antennal (An), five median (M) and eight sutural (S) mac (Fig. 5). Prelabral and labral chaetae as 4/5, 5, 4, all smooth, a0, a1 longer than a2; labral papillae absent (Fig. 6). Basal chaeta on maxillary outer lobe almost as thick as apical one; sublobal plate with three smooth chaetae-like processes (Fig. 7). Lateral process (l. p.) of labial palp E differentiated, as thick as normal chaeta, with tip almost reaching apex of papilla E (Fig. 8). Labial base with MM_1_R_1_ReL_1_L_2_, chaeta e smooth and other ciliate, R_1_ sometimes absent, R 0.60–0.73 length of M; anterior post-labial chaetae slightly expanded (Figs 9–11).

**Figures 12–17.**
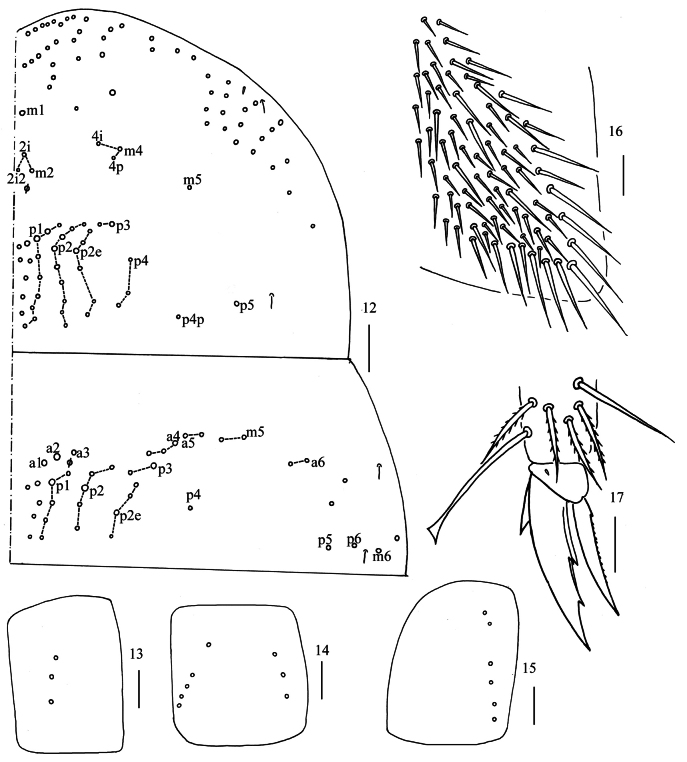
*Homidialongiantenna* sp. nov. **12** chaetotaxy of Th. II−III (right side) **13−15** coxal chaetotaxy of fore, middle and hind leg **16** trochanteral organ **17** hind foot complex (lateral view). Scale bars: 50 μm (**12**); 20 μm (**13−17**).

**Figure 18.**
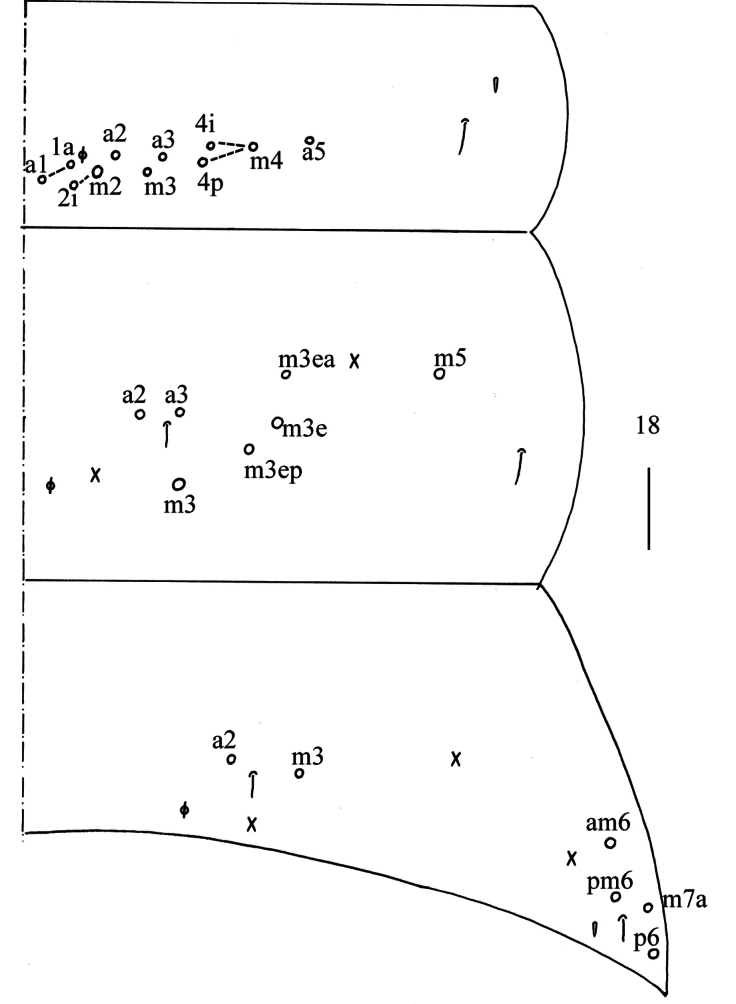
Chaetotaxy of Abd. I−III of *Homidialongiantenna* sp. nov. (right side). Scale bar: 50 μm.

**Figure 19.**
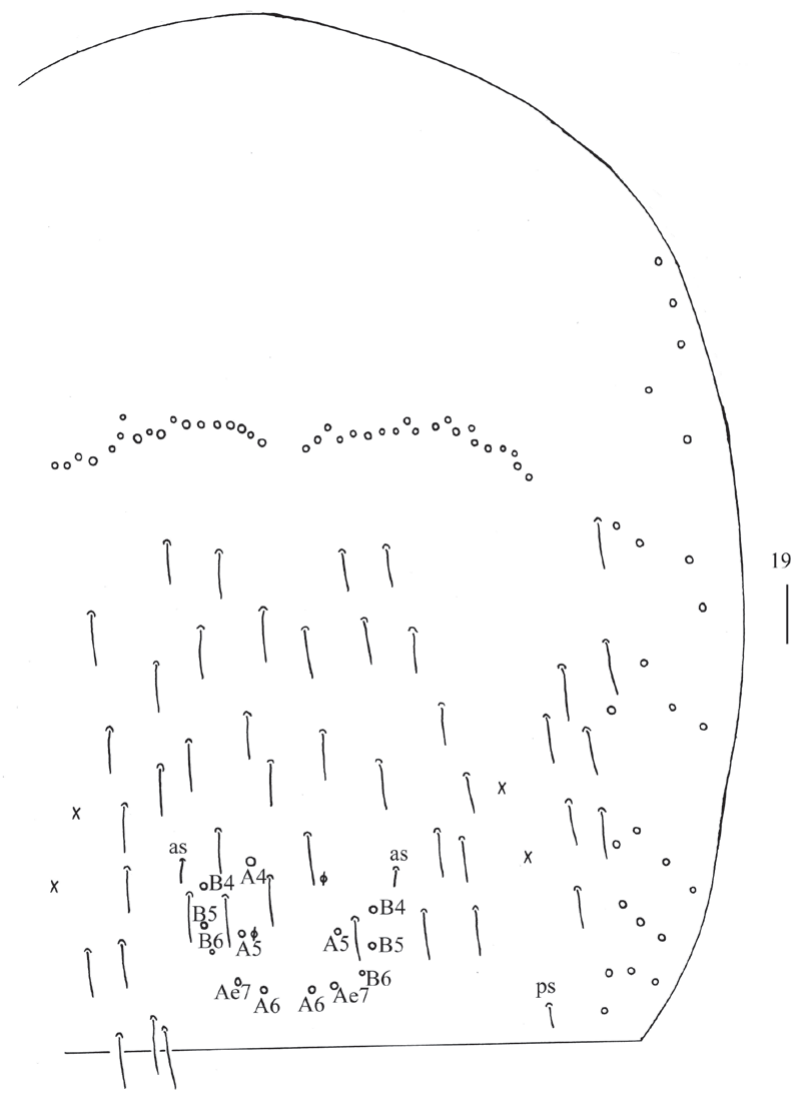
Chaetotaxy of Abd. IV of *Homidialongiantenna* sp. nov. (right side). Scale bar: 50 μm.

**Figures 20–27.**
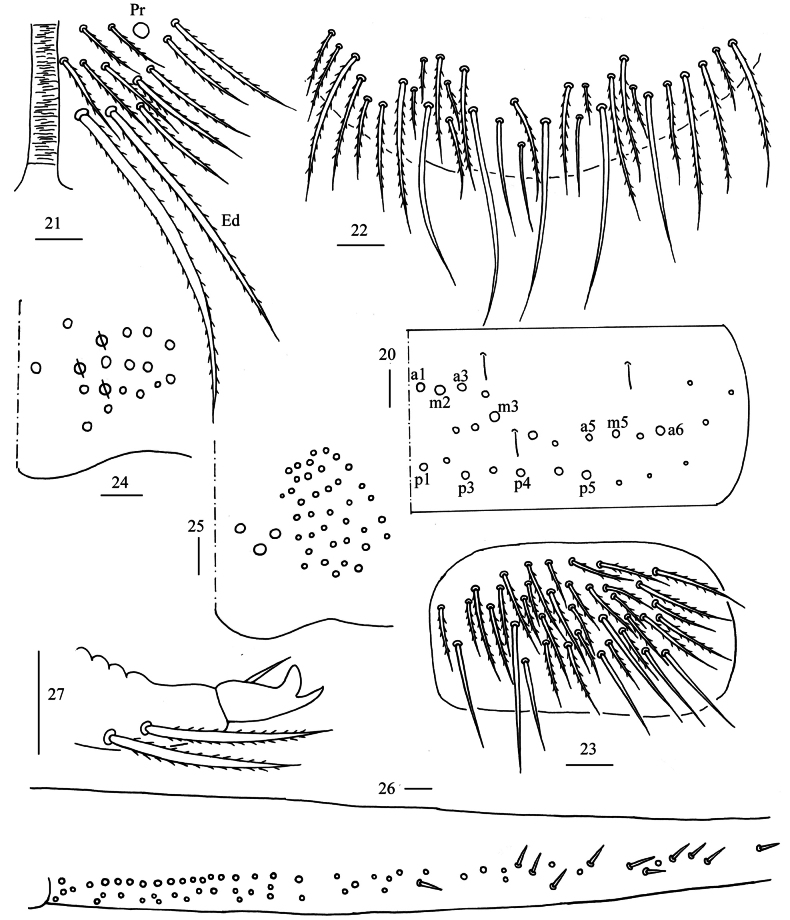
*Homidialongiantenna* sp. nov. **20** chaetotaxy of Abd. V (right side) **21** anterior face of ventral tube apically **22** posterior face of ventral tube apically **23** lateral flap of ventral tube **24** manubrial plaque (dorsal view) **25** ventro-apical part of manubrium **26** proximal section of dens (circles also representing spines) **27** mucro. Scale bars: 20 μm.

***Thorax*.** Tergal ms formula on Th. II–Abd. V as 1, 0/1, 0, 1, 0, 0, sens as 2, 2/1, 2, 2, 23, 3 (Figs 12, 18–20). Th. II with four medio-medial (m1, m2, m2i, m2i2), three medio-sublateral (m4, m4i, m4p), 30–38 posterior mac. Th. III with about 39–41 mac (Fig. 12). Coxal macrochaetal formula as 3/4+1, 3/4+2 (Figs 13–15). Trochanteral organ with 71–76 smooth chaetae (Fig. 16). Tenent hair clavate, 0.95–0.98 length of inner edge of unguis; unguis with three inner teeth, basal pair located at 0.38–0.39 distance from base of inner edge of unguis, unpaired tooth at 0.62–0.64 distance from base; unguiculus lanceolate, outer edge slightly serrate (Fig. 17).

***Abdomen*.** Range of Abd. IV length as 9.00–9.30 times as dorsal axial length of Abd. III. Abd. I with 11 (a1a, a1–3, m2i, m2–4, m4i, m4p and a5) mac. Abd. II with six (a2, a3, m3, m3e, m3ea, m3ep) central, one (m5) lateral mac. Abd. III with two (a2, m3) central, four (am6, pm6, m7a, p6) lateral mac (Fig. 18). Abd. IV with two (as, ps) normal sens, 14–20 anterior, 6–7 (A4–6, B4–6, Ae7, A4 sometimes absent) posterior and 24–26 lateral mac (Fig. 19). Abd. V with three sens (Fig. 20). Anterior face of ventral tube not seen entirely, line connecting proximal (Pr) and external-distal (Ed) mac oblique to median furrow (Fig. 21); posterior face with five or eight distal smooth and numerous ciliate chaetae (Fig. 22); lateral flap with 7–8 smooth and 19–30 ciliate chaetae (Fig. 23). Manubrial plate dorsally with 14–15 ciliate mac and 3(2) pseudopores (Fig. 24); ventrally with 33–41 ciliate chaetae on each side (Fig. 25). Dens with 54–78 smooth inner spines (Fig. 26). Mucro bidentate with subapical tooth larger than apical one; tip of basal spine reaching apex of subapical tooth; distal smooth section of dens almost equal to mucro in length (Fig. 27).

##### Etymology.

Named after its characteristic long antennae.

##### Ecology.

Found in the leaf litter.

##### Remarks.

The new species is characterised by the long antennae and the slightly expanded post-labial chaetae, and can be easily distinguished from all known species of *Homidia*. It is similar to the species *H.apigmenta* Shi, Pan & Zhang, 2010, *H.pseudofascia* Pan, Zhang & Li, 2015, and *H.wanensis* Pan & Ma, 2021 in the expanded post-labial chaetae and colour pattern, but can be separated from them by the long antennae and other characters. It is also similar to *H.jordanai* Pan, Shi & Zhang, 2011 in the long antennae, but significant differences exist between them, such as the post-labial chaetotaxy and central mac on Abd. III and other characters. The detailed character comparisons are listed in Table 2.

**Table 2.** Main differences among the new species and related species of *Homidia*.

Characters	*Homidialongiantenna* sp. nov.	*Homidiaapigmenta*	*Homidiajordanai*	*Homidiapseudofascia*	*Homidiawanensis*
Length ratio of antenna to body	0.98–1.07	0.50	about 1.00	0.59–0.67	0.60–0.62
An irregular transverse stripe on Abd. IV posteriorly	present	absent	absent	present	present
Chaetal formula of labial base	MM_1_(R_1_)ReL_1_L_2_	M(M_1_)ReL_1_L_2_, L_1_ & L_2_ expanded	MReL_1_L_2_	MM_1_ReL_1_L_2_	MReL_1_L_2_
Anterior post-labial chaetae	slightly expanded	strong expanded	not expanded	slightly expanded	slightly expanded
Inner teeth on unguis	3	4	4	4	4
Mac on Abd. IV anteriorly	14–20	6–9	6–9	8–11	12–13
Mac on Abd. IV posteriorly	6–7	5	3(4)	7–9	7–9
Dental spines	54–78	18–39	20–40	36–50	83

#### ﻿ Homidia guangxiensis sp. nov.

Taxon classificationAnimaliaCollembolaEntomobryidae

126A2CC1-4FBE-52B1-9AA2-2AC1211FC973

https://zoobank.org/D3D8DC59-3275-448D-8E92-B82F205AF05B

Figs 28–29 , 30−36 , 37−39 , 40−45 , 46 , 47 , 48−51 , 52–56 , Table 3

##### Type material.

***Holotype*** • ♀ on slide, China, Guangxi Zhuang Autonomous Region, Guilin City, Longsheng Autonomous County, Huaping Natural Reserve, Tianping Mountain, Power Station, 2-VI-2023, 25°37′40″N, 109°54′19″E, 682.0 m asl, sample number 1283. ***Paratypes*** • 3♀♀ on slides, same data as holotype • ♀ on slide, China, Guangxi Zhuang Autonomous Region, Guilin City, Longsheng Autonomous County, Huaping Natural Reserve, Tianping Mountain, 31-V-2023, 25°37′52″N, 109°54′47″E, 935.4 m asl, sample number 1281. All collected by Y-T Ma.

##### Description.

***Size*.** Body length up to 2.86 mm.

**Figures 28, 29.**
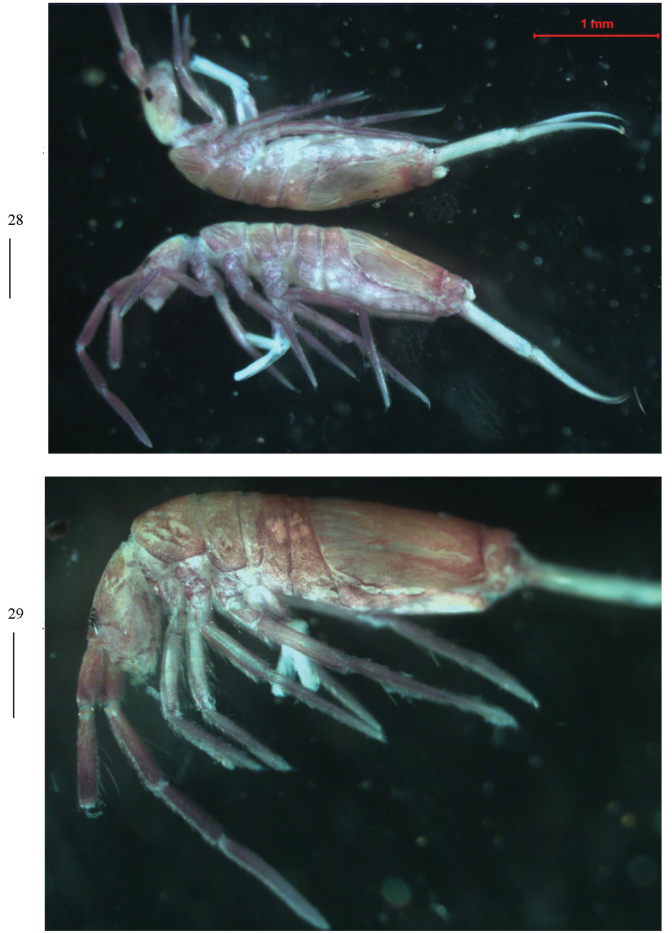
Habitus of *Homidiaguangxiensis* sp. nov. (lateral view). Scale bars: 500 μm.

***Coloration*.** Ground colour pale white to yellow; eye patches dark blue; brown to blue-violet pigment present on whole dorsal body, antennae, legs, ventral tube, and manubrium. Some unpigmented irregular stripes or spots present on dorsal side of body (Figs 28, 29).

**Figures 30–36.**
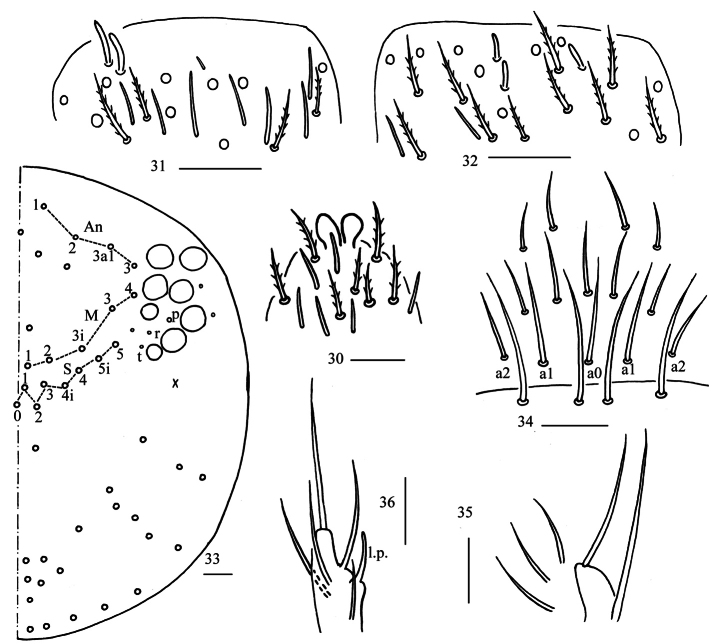
*Homidiaguangxiensis* sp. nov. **30** apex of Ant. IV (dorsal view) **31** distal Ant. III (ventral view) **32** distal Ant. II (ventral view) **33** dorsal head (right side) **34** prelabrum and labrum **35** maxillary palp and outer lobe (right side) **36** labial palp. Scale bars: 20 μm.

**Figures 37–39.**
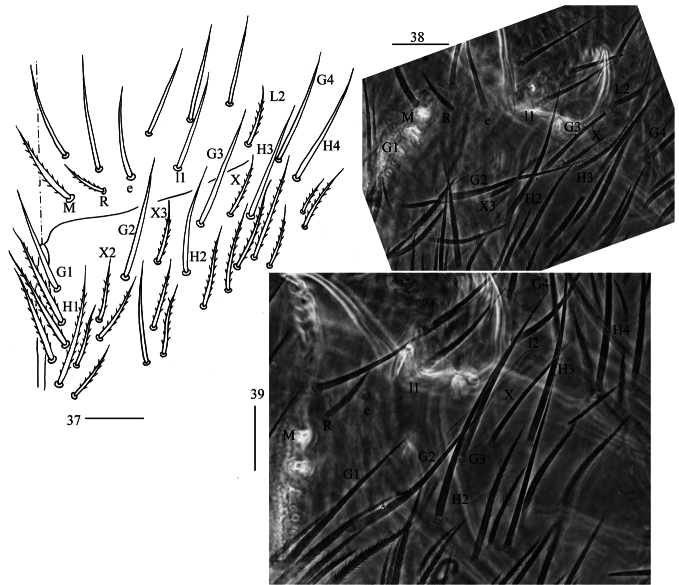
*Homidiaguangxiensis* sp. nov. **37** labial and post-labial chaetotaxy (right side) **38, 39** photographs of labial and post-labial chaetotaxy (right side). Scale bars: 20 μm.

***Head*.** Antenna not annulated and 0.60–0.80 times length of body. Ratio of Ant. I–IV as 1.00/1.27–1.70/1.21–1.40/1.67–2.41. Distal part of Ant. IV with many sensory chaetae and normal ciliate chaetae, apical bulb bilobed (Fig. 30). Sensory organ of Ant. III with two rod-like chaetae (Fig. 31). Sensory organ of Ant. II with 3–4 rod-like chaetae (Fig. 32). Eyes 8+8, G and H smaller than others, interocular chaetae as p, r, t mes. Dorsal chaetotaxy of head with four antennal (An), five median (M) and eight sutural (S) mac (Fig. 33). Prelabral and labral chaetae as 4/5, 5, 4, all smooth, a0, a1 longer than a2; labral papillae absent (Fig. 34). Basal chaeta on maxillary outer lobe slightly thicker than as apical one; sublobal plate with three smooth chaetae-like processes (Fig. 35). Lateral process (l. p.) of labial palp E differentiated, as thick as normal chaeta, with tip almost reaching apex of papilla E (Fig. 36). Labial base with MRel_1_L_2_, M sometimes smooth, R ciliate and 0.50–0.53 length of M, chaetae e and l_1_ smooth, L_2_ rarely smooth; some post-labial chaetae (G_1–4_, H_2–4_, sometimes X and an unnamed chaeta) smooth (Figs 37–39).

**Figures 40–45.**
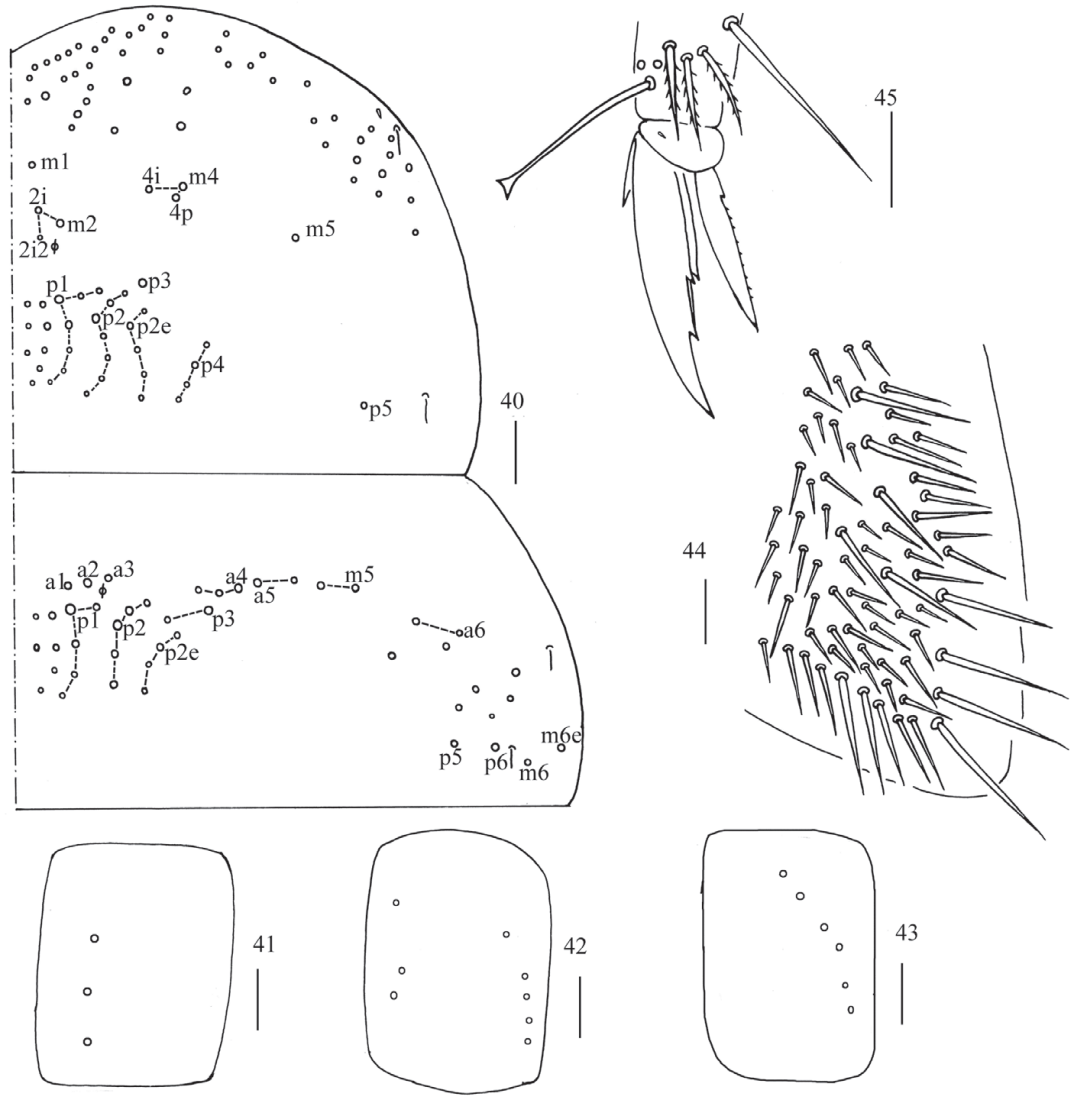
*Homidiaguangxiensis* sp. nov. **40** chaetotaxy of Th. II−III (right side) **41−43** coxal chaetotaxy of fore, middle and hind leg **44** trochanteral organ **45** hind foot complex (lateral view). Scale bars: 50 μm (**40**); 20 μm (**41−45**).

**Figure 46.**
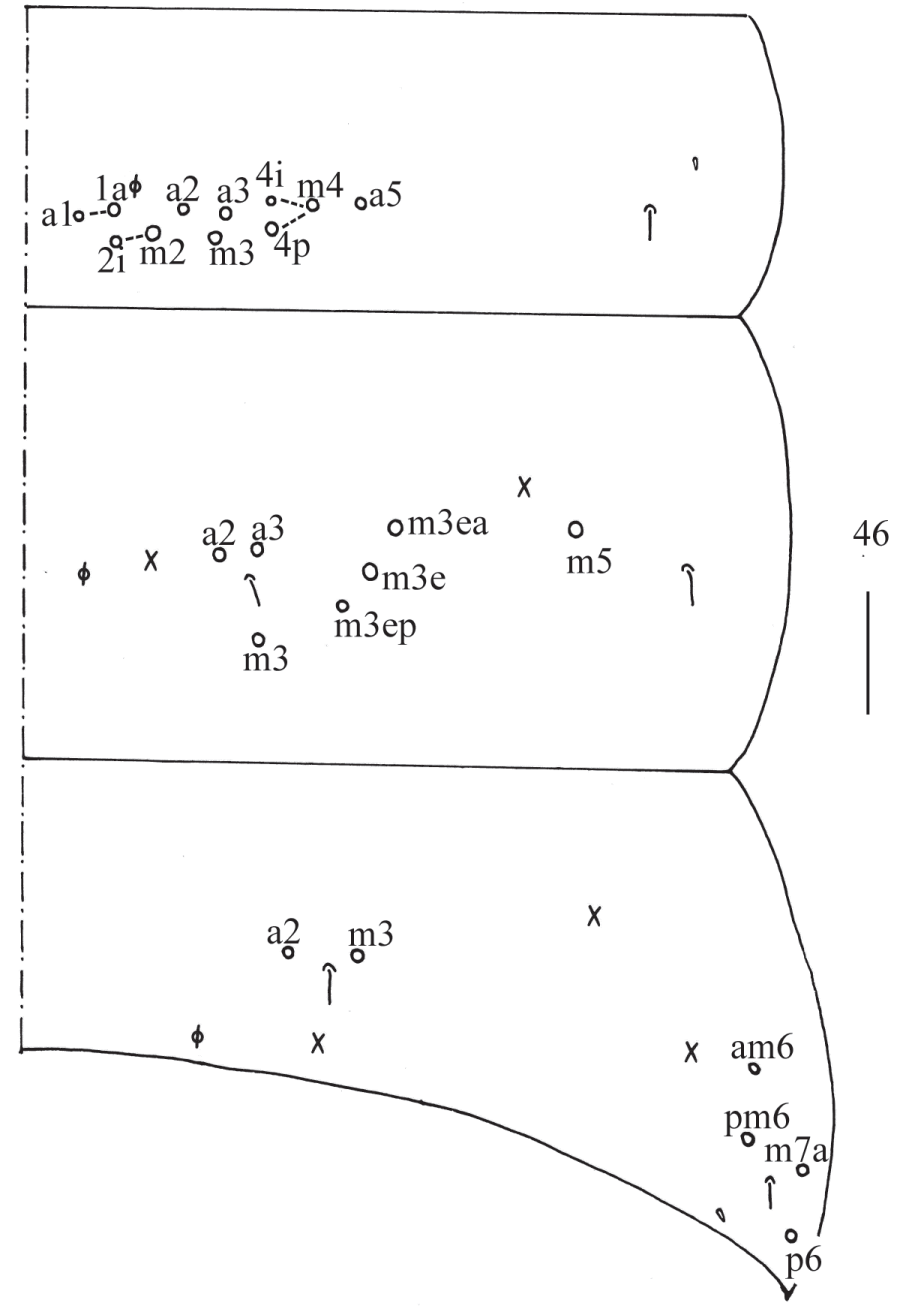
Chaetotaxy of Abd. I−III of *Homidiaguangxiensis* sp. nov. (right side) Scale bar: 50 μm.

**Figure 47.**
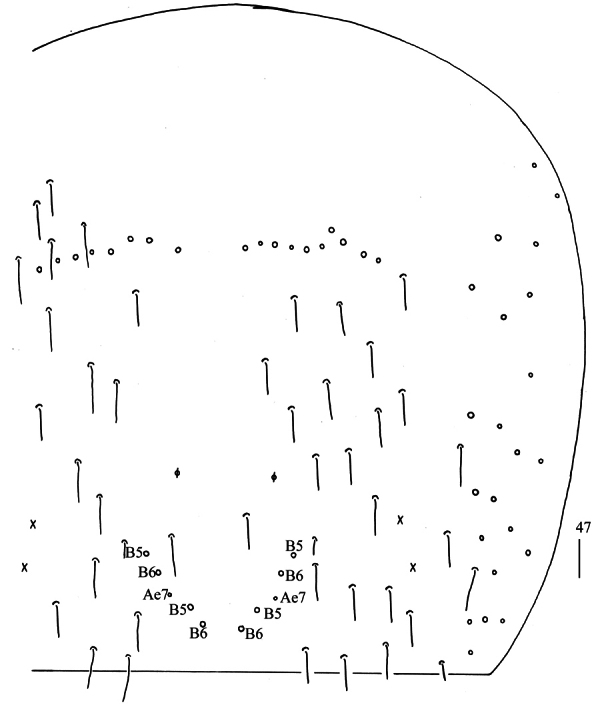
Chaetotaxy of Abd. IV of *Homidiaguangxiensis* sp. nov. (right side). Scale bar: 50 μm.

**Figures 48–51.**
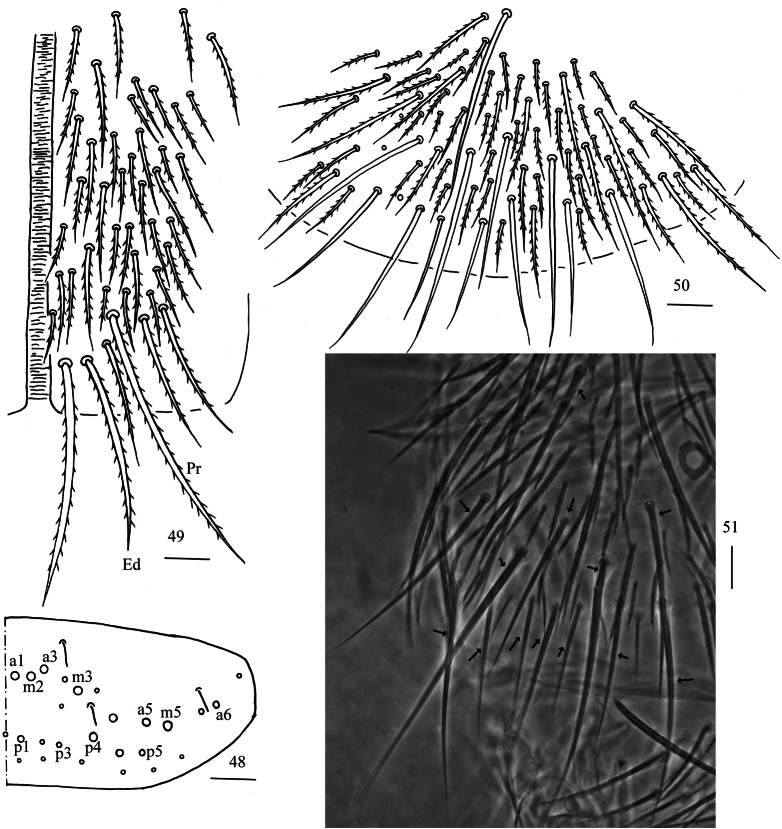
*Homidiaguangxiensis* sp. nov. **48** chaetotaxy of Abd. V (right side) **49** anterior face of ventral tube **50** posterior face of ventral tube apically **51** photomicrograph of posterior face of ventral tube apically. Scale bars: 20 μm.

***Thorax*.** Tergal ms formula on Th. II–Abd. V as 1, 0/1, 0, 1, 0, 0, sens as 2, 2/1, 2, 2, 18–36, 3 (Figs 40, 46–48). Th. II with four medio-medial (m1, m2, m2i, m2i2), three medio-sublateral (m4, m4i, m4p), 33–39 posterior mac. Th. III with 44–49 mac (Fig. 40). Coxal macrochaetal formula as 3/4+1, 3/4+2 (Figs 41–43). Trochanteral organ with 44–71 smooth chaetae (Fig. 44). Tenent hair clavate, 0.68–0.88 length of inner edge of unguis; unguis with three inner teeth, basal pair located at 0.32–0.40 distance from base of inner edge of unguis, unpaired tooth at 0.59–0.68 distance from base; unguiculus lanceolate, outer edge slightly serrate (Fig. 45).

**Figures 52–56.**
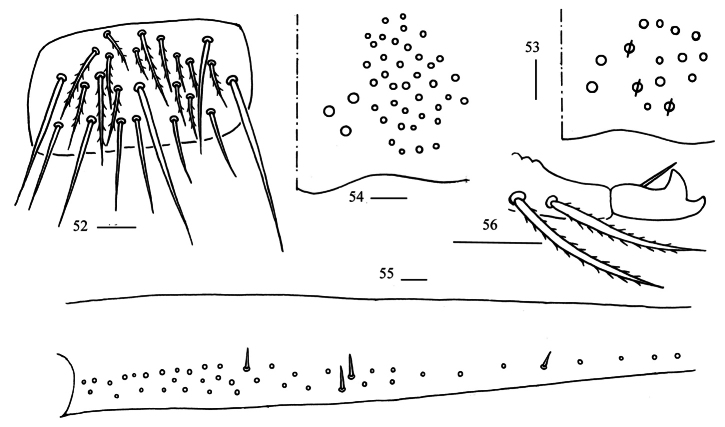
*Homidiaguangxiensis* sp. nov. **52** lateral flap of ventral tube **53** manubrial plaque (dorsal view) **54** ventro-apical part of manubrium **55** proximal section of dens (circles also representing spines) **56** mucro. Scale bars: 20 μm.

***Abdomen*.** Range of Abd. IV length as 6.51–8.75 times as dorsal axial length of Abd. III. Abd. I with 11 (rarely 10) (a1–3, m2i, m2–4, m4i, m4p and a5, a1a rarely absent) mac. Abd. II with six (a2, a3, m3, m3e, m3ea, m3ep) central, one (m5) lateral mac. Abd. III with two (a2, m3) central, four (am6, pm6, m7a, p6) lateral mac (Fig. 46). Abd. IV with two (as, ps) normal sens, 8–11 anterior, five (A5–6, B4–6, Ae7) posterior and 20–23 lateral mac (Fig. 47). Abd. V with three sens (Fig. 48). Anterior face of ventral tube with 44–46 ciliate chaetae on each side, line connecting proximal (Pr) and external-distal (Ed) mac oblique to median furrow (Fig. 49); posterior face with 9–18 smooth and numerous ciliate chaetae (Figs 50, 51); lateral flap with 7–12 (19) smooth and 11–19 ciliate chaetae (Fig. 52). Manubrial plate dorsally with 10–14 ciliate mac and three pseudopores (Fig. 53); ventrally with (26) 40–47 ciliate chaetae on each side (Fig. 54). Dens with 24–48 smooth inner spines (Fig. 55). Mucro bidentate with subapical tooth larger than apical one; tip of basal spine reaching apex of subapical tooth; distal smooth section of dens almost equal to mucro in length (Fig. 56).

##### Etymology.

Named after its locality: Guangxi Zhuang Autonomous Region.

##### Ecology.

Found in the leaf litter.

##### Remarks.

The new species can be easily distinguished from other species of the genus by the smooth post-labial chaetae and the number and location of smooth chaetae on the posterior face of the ventral tube. Among the known *Homidia* species, except those species with expanded post-labial chaetae, the post-labial chaetae are rarely mentioned because most have normal ciliate chaetae. Smooth post-labial chaetae are discovered for the first time in the genus. In addition, the smooth chaetae on the posterior face of the ventral tube are usually located at the most distal part of the ventral tube and their number is usually less than 10 in the genus. However, the number and location of the smooth chaetae on the posterior face of the new species are peculiar. It is similar to the species *H.acutus* Jing & Ma, 2022, *H.pseudozhangi* Jing & Ma, 2023 and *H.zhangi* Pan & Shi, 2012 in the colour pattern, but can be separated from them by the smooth post-labial chaetae, inner teeth on unguis and other characters. The detailed character comparisons are listed in Tables 3, 4.

**Table 3.** Variation in some characters of the species described in the present paper.

Species	Specimen number	Mac on Abd. I	Anterior mac on Abd. IV	Posterior mac on Abd. IV	Lateral mac on Abd. IV	Tip of tenent hair	Smooth post-labial mac	Smooth mac on posterior face of ventral tube
*Homidialongiantenna* sp. nov.	1281-3A	11+11	14+15	6+7	25+25	clavate	absent	?
	1281-3B	11+11	18+20	6+7	24+26	clavate	absent	8
*Homidiaguangxiensis* sp. nov.	1281-13	10+11	10+10	5+5	20+22	clavate	present	10
	1283-8A	11+11	10+?	5+5	?+?	clavate	present	9
	1283-8B	11+12	9+10	5+5	23+?	clavate	present	12
	1283-9A	11+11	8+10	5+5	23+?	clavate	preent	17
	1283-9B	11+11	9+11	5+5	20+22	clavate	present	18
*Homidiahuapingensis* sp. nov.	1274-2B	11+11	10+11	5+5	19+20	point	present	?
	1274-2C	11+12	10+11	5+6	20+20	point	present	9
	1279-6B	11+12	11+12	6+6	21+?	point	present	6
	1281-10A	11+11	9+10	5+6	19+20	point	present	9
	1281-10B	11+11	9+9	5+5	22+22	point	present	8
	1283-8C	12+?	9+10	5+6	22+23	point	present	5
*Homidiaoligoseta* sp. nov.	1281-11A	11+11	3+5	4+4	16+17	point	?	7
	1281-11B	11+?	3+3	4+5	16+16	point	absent	7
	1281-11C	10+10	3+4	4+4	16+17	point	absent	7
	1282-4A	11+11	3+4	4+4	15+?	point	absent	5
	1282-4B	11+11	3+3	4+4	12+?	point	absent	5
	1282-5	11+11	3+3	4+4	13+?	point	absent	5
	1282-6	11+11	3+3	4+4	13+15	point	absent	6
	1283-1A	11+11	3+3	4+4	13+13	point	absent	5
	1283-1B	11+11	3+4	4+4	13+?	point	absent	5
	1283-2A	10+11	4+5	4+4	12+14	point	absent	5
	1283-2B	10+11	3+3	4+4	11+?	point	absent	?
	1283-3	10+11	3+3	4+4	13+14	point	absent	5
	1283-10A	11+11	4+4	4+4	14+?	point	absent	5
	1283-10B	11+11	4+4	4+5	13+13	point	absent	5
*Homidiaacutus*	1229-1A	11+11	6+6	5+5	15+?	point	present	6
	1229-1B	11+?	6+6	5+5	1516	point	present	6
	1229-2A	11+11	?+?	5+5	16+?	point	present	6
	1229-2B	11+11	6+6	5+5	16+16	point	present	?

*? not clearly seen.

**Table 4.** Main differences among the three new species and related species of *Homidia*.

Characters	*Homidiaguangxiensis* sp. nov.	*Homidiahuapingensis* sp. nov.	*Homidiaoligoseta* sp. nov.	*Homidiaacutus*	*Homidiapseudozhangi*	*Homidiazhangi*
Medial stripe on Th. II–III	absent	absent	absent	absent	present	absent
Smooth post-labial chaetae	present	present	absent	present	absent	absent
Tenent hair	clavate	pointed	pointed	pointed	clavate	clavate
Inner teeth on unguis	3	3	3	3	4	4
Smooth chaetae on posterior face of ventral tube	9–18	5–9	5–7	6	4–5	4
Relative position of ms to sens on Abd. I	antero-external	antero-external	antero-external	antero-external	antero-external	antero-internal
Relative position of middle sens to m3 on Abd. V	postero-external	postero-external	postero-external	postero-external	antero-external	postero-external
Mac on Abd. IV anteriorly	8–11	9–12	3–4(5)	6	7–12	8–10
Mac on Abd. IV posteriorly	5	5–6	4(5)	5	6	3(4)

#### ﻿ Homidia huapingensis sp. nov.

Taxon classificationAnimaliaCollembolaEntomobryidae

58778C64-83F5-59A0-87D3-0120DB65801F

https://zoobank.org/164C2C44-EE5F-40AF-B682-D01ACA2BC432

Figs 57–58 , 59−65 , 66−68 , 69−76 , 77 , 78 , 79–86 , Tables 3 , 4

##### Type material.

***Holotype*** • ♀ on slide, China, Guangxi Zhuang Autonomous Region, Guilin City, Longsheng Autonomous County, Huaping Natural Reserve, Tianping Mountain, 31-V-2023, 25°37′52″N, 109°54′47″E, 935.4 m asl, sample number 1281. ***Paratypes*** • 2♀ on slides, China, Guangxi Zhuang Autonomous Region, Guilin City, Longsheng Autonomous County, Huaping Natural Reserve, Guangfu Mountain, 26-V-2023, 25°33′44″N, 109°56′16″E, 1341.0 m asl, sample number 1274 • ♀ on slide, China, Guangxi Zhuang Autonomous Region, Guilin City, Longsheng Autonomous County, Huaping Natural Reserve, Guangfu Mountain, 29-V-2023, 25°33′25″N, 109°56′38″E, 1340.5 m asl, sample number 1279 • ♀ on slide, China, Guangxi Zhuang Autonomous Region, Guilin City, Longsheng Autonomous County, Huaping Natural Reserve, Tianping Mountain, 31-V-2023, 25°37′52″N, 109°54′47″E, 935.4 m asl, sample number 1281 • 2♀ on slides, China, Guangxi Zhuang Autonomous Region, Guilin City, Longsheng Autonomous County, Huaping Natural Reserve, Tianping Mountain, Power Station, 2-VI-2023, 25°37′40″N, 109°54′19″E, 682.0 m asl, sample number 1283. All collected by Y-T Ma.

##### Description.

***Size*.** Body length up to 2.92 mm.

**Figures 57, 58.**
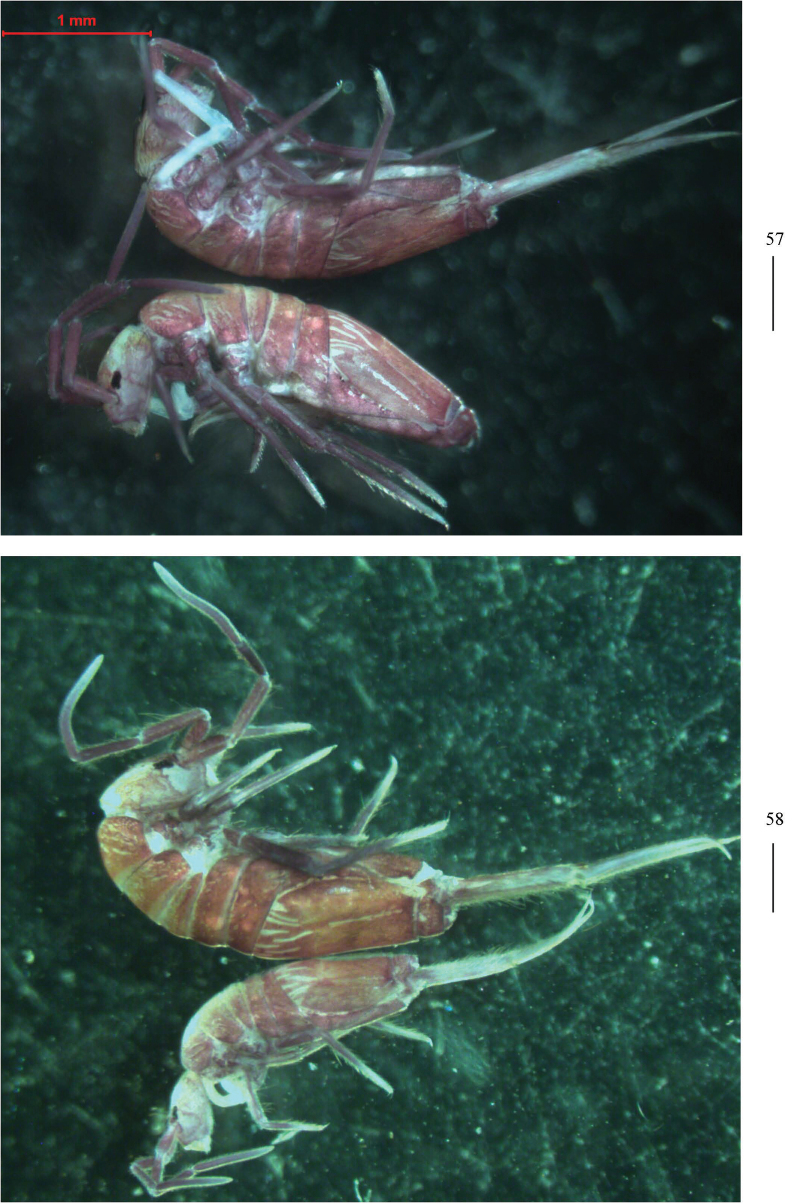
Habitus of *Homidiahuapingensis* sp. nov. (lateral view). Scale bars: 500 μm.

***Coloration*.** Ground colour pale white to yellow; eye patches dark blue; brown to blue-violet pigment present on whole dorsal body, antennae, legs, ventral tube, and manubrium. Some unpigmented irregular stripes or spots present on dorsal side of body (Figs 57, 58).

**Figures 59–65.**
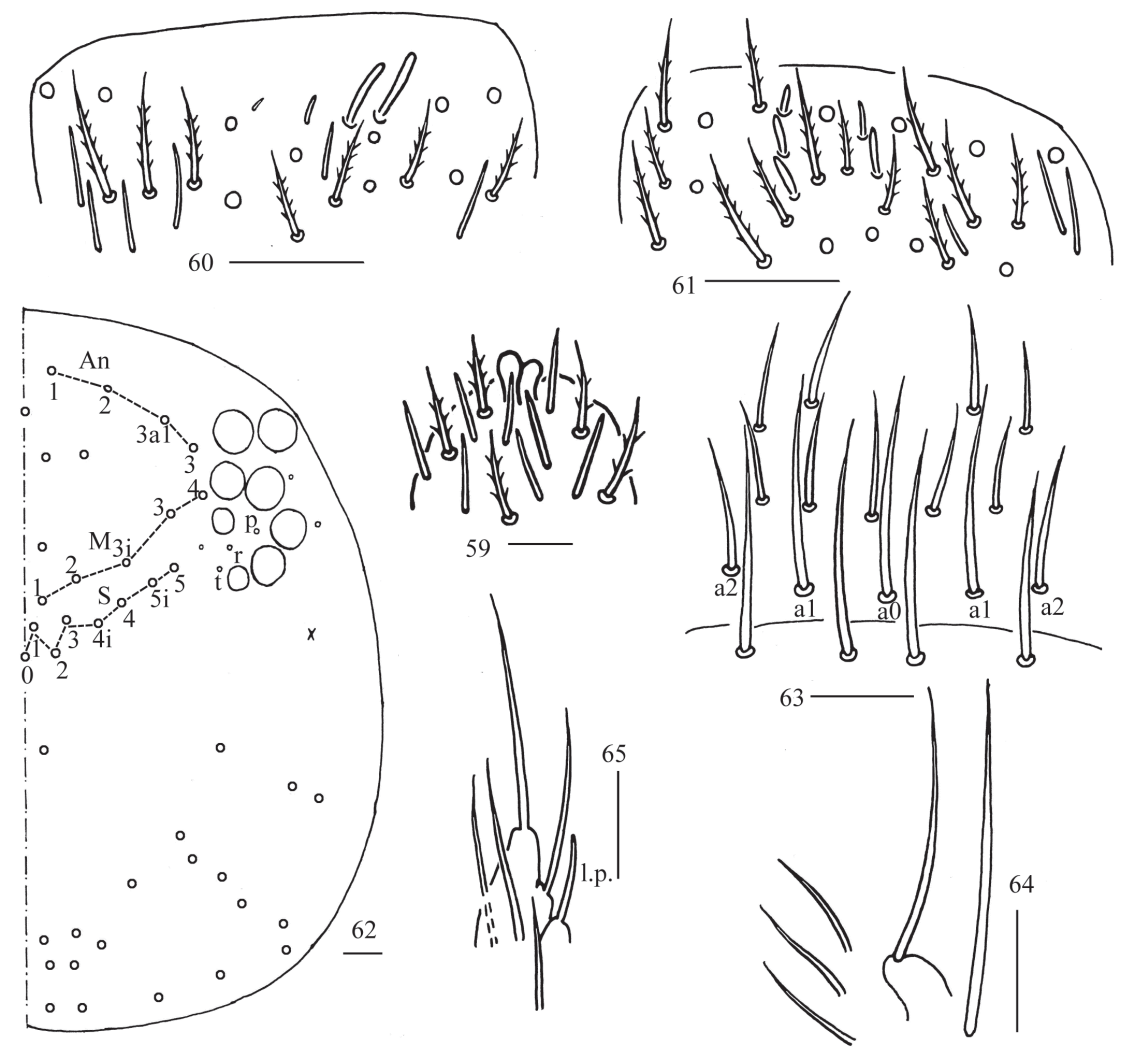
*Homidiahuapingensis* sp. nov. **59** apex of Ant. IV (dorsal view) **60** distal Ant. III (ventral view) **61** distal Ant. II (ventral view) **62** dorsal head (right side) **63** prelabrum and labrum **64** maxillary palp and outer lobe (right side) **65** labial palp. Scale bars: 20 μm.

**Figures 66–68.**
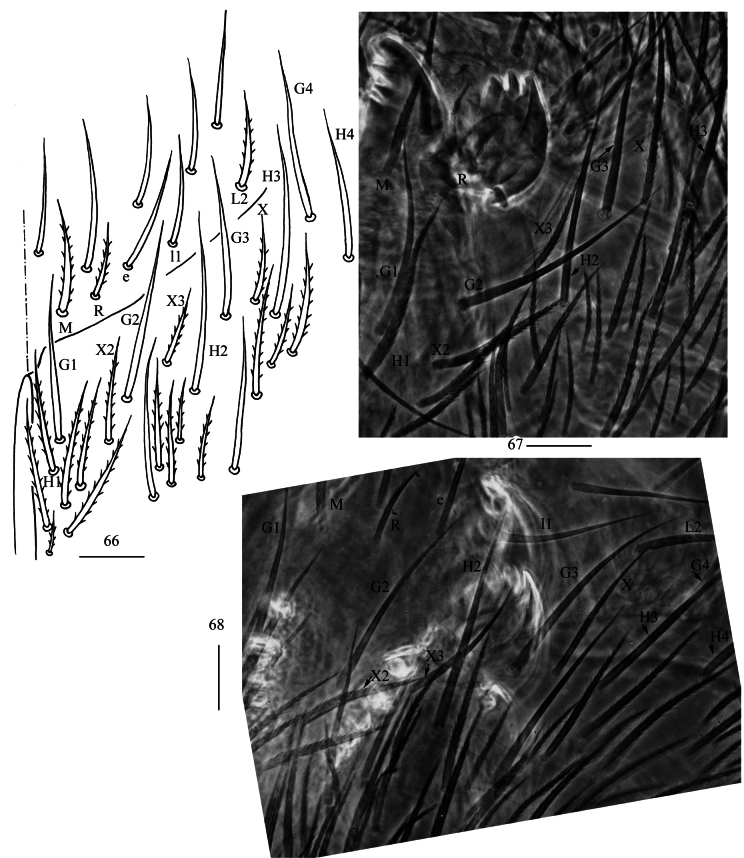
*Homidiahuapingensis* sp. nov. **66** labial and post-labial chaetotaxy (right side) **67, 68** photographs of labial and post-labial chaetotaxy (right side). Scale bars: 20 μm.

***Head*.** Antenna not annulated and 0.59–0.64 times length of body. Ratio of Ant. I–IV as 1.00/1.28–1.40/1.11–1.36/2.00–2.25. Distal part of Ant. IV with many sensory chaetae and normal ciliate chaetae, apical bulb bilobed (Fig. 59). Sensory organ of Ant. III with two rod-like chaetae (Fig. 60). Sensory organ of Ant. II with 4–5 rod-like chaetae (Fig. 61). Eyes 8+8, G and H smaller than others, interocular chaetae as p, r, t mes. Dorsal chaetotaxy of head with four antennal (An), five median (M) and eight sutural (S) mac (Fig. 62). Prelabral and labral chaetae as 4/5, 5, 4, all smooth, a0, a1 longer than a2; labral papillae absent (Fig. 63). Basal chaeta on maxillary outer lobe slightly thicker than as apical one; sublobal plate with three smooth chaetae-like processes (Fig. 64). Lateral process (l. p.) of labial palp E differentiated, as thick as normal chaeta, with tip almost reaching apex of papilla E (Fig. 65). Labial base with MRel_1_L_2_, M rarely smooth, R ciliate and 0.50–0.69 length of M, chaetae e and l_1_ smooth, L_2_ ciliate; some post-labial chaetae (G_1–4_, H_2–4_, rarely X, X_3_ and 1–2 unnamed chaetae) smooth (Figs 66–68).

**Figures 69–76.**
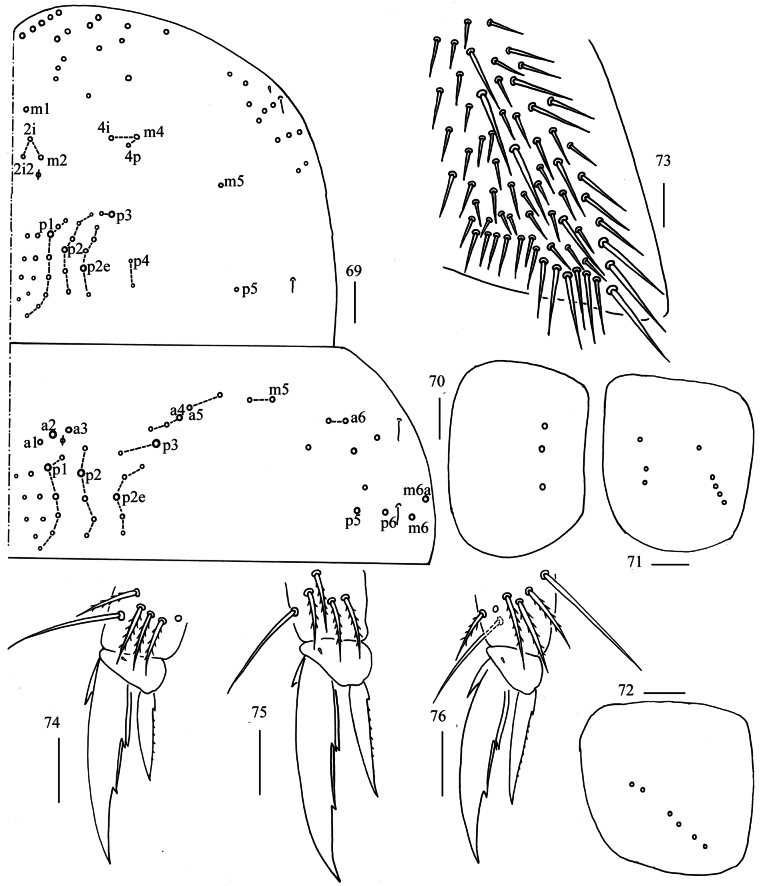
*Homidiahuapingensis* sp. nov. **69** chaetotaxy of Th. II−III (right side) **70−72** coxal chaetotaxy of fore, middle and hind leg **73** trochanteral organ **74−76** foot complex of fore, middle and hind leg (lateral view). Scale bars: 50 μm (**69**); 20 μm (**70−76**).

**Figure 77.**
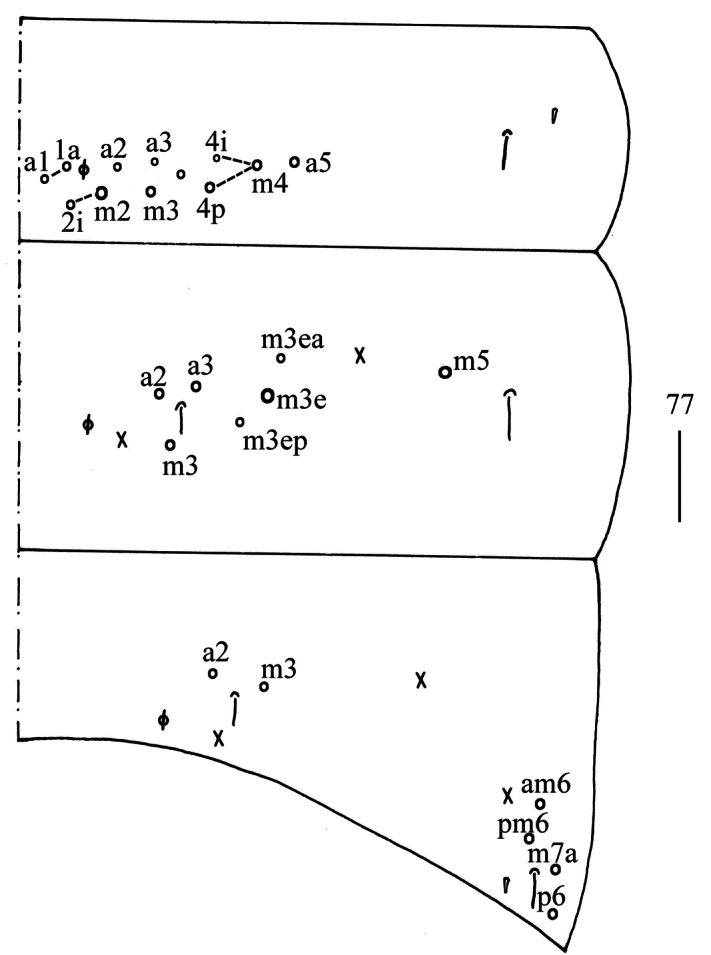
Chaetotaxy of Abd. I−III of *Homidiahuapingensis* sp. nov. (right side). Scale bar: 50 μm.

**Figure 78.**
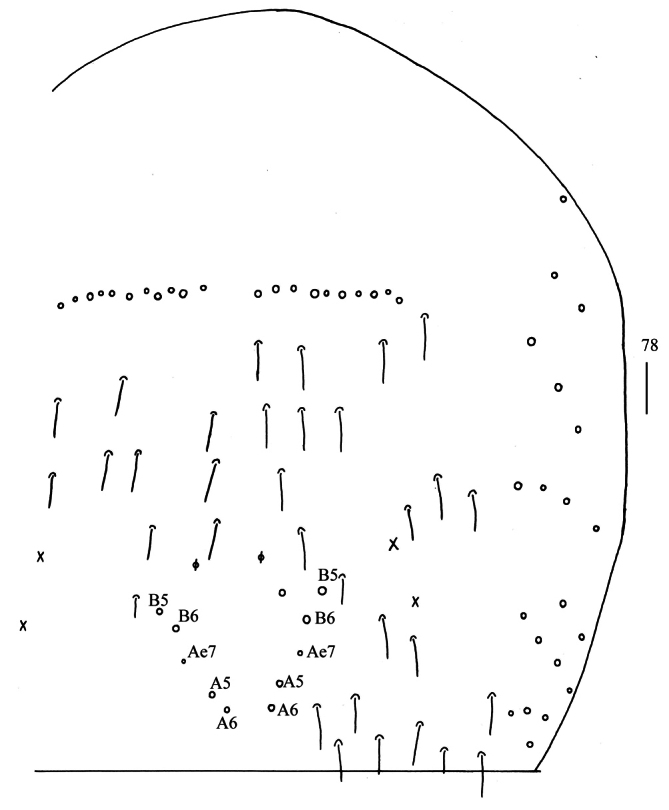
Chaetotaxy of Abd. IV of *Homidiahuapingensis* sp. nov. (right side). Scale bar: 50 μm.

**Figures 79–86.**
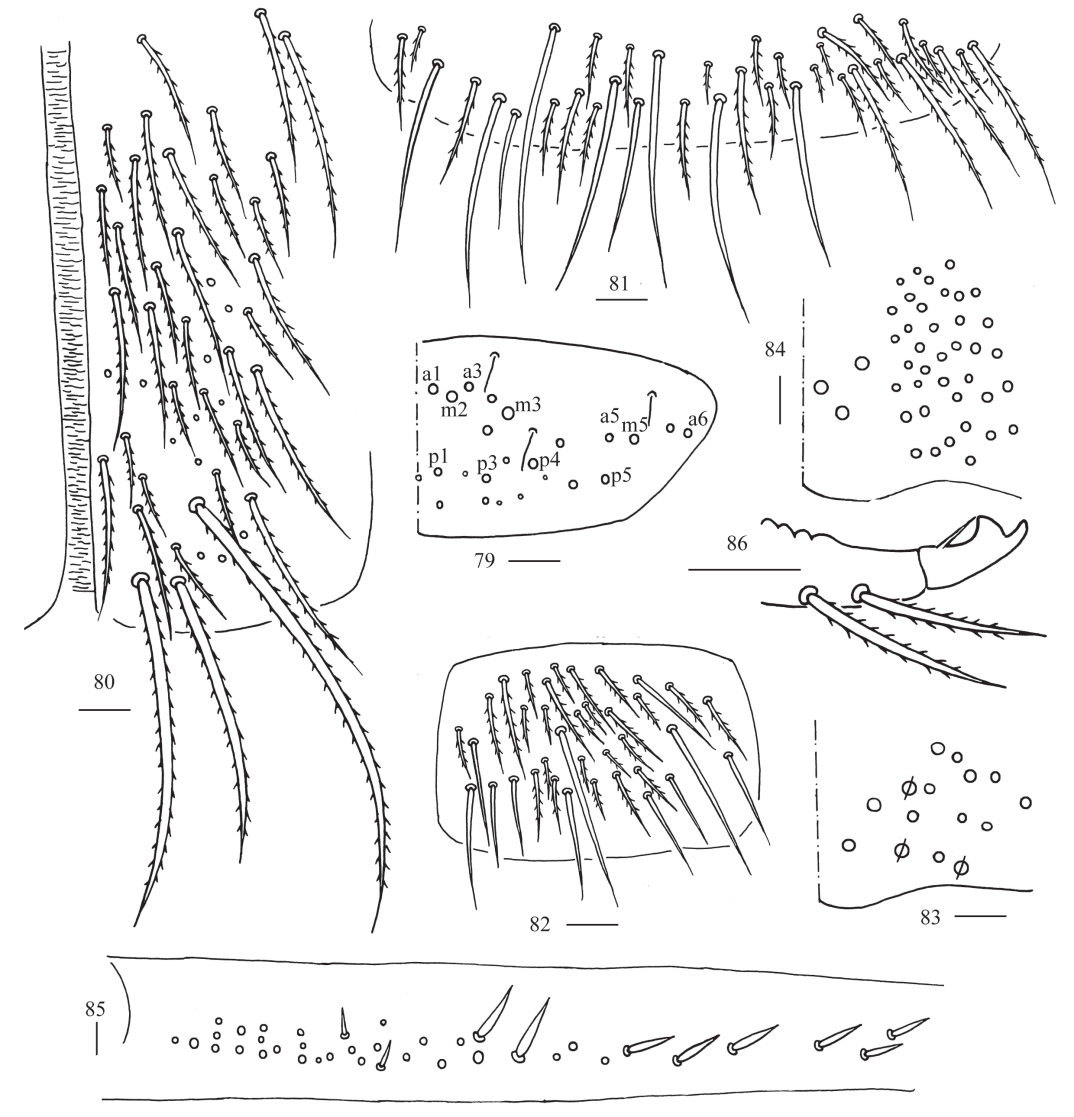
*Homidiahuapingensis* sp. nov. **79** chaetotaxy of Abd. V (right side) **80** anterior face of ventral tube apically **81** posterior face of ventral tube apically **82** lateral flap of ventral tube **83** manubrial plaque (dorsal view) **84** ventro-apical part of manubrium **85** proximal section of dens (circles also representing spines) **86** mucro. Scale bars: 20 μm.

***Thorax*.** Tergal ms formula on Th. II–Abd. V as 1, 0/1, 0, 1, 0, 0, sens as 2, 2/1, 2, 2, 23–37, 3 (Figs 69, 77–79). Th. II with four medio-medial (m1, m2, m2i, m2i2), three medio-sublateral (m4, m4i, m4p), 32–42 posterior mac. Th. III with 45–57 mac (Fig. 69). Coxal macrochaetal formula as 3/4+1, 3/4+2 (Figs 70–72). Trochanteral organ with about 40–70 smooth chaetae (Fig. 73). All tenent hairs pointed and 0.53–0.68 length of inner edge of unguis; unguis with three inner teeth, basal pair located at 0.29–0.35 distance from base of inner edge of unguis, unpaired tooth at 0.59–0.62 distance from base; unguiculus lanceolate, outer edge slightly serrate (Figs 74–76).

***Abdomen*.** Range of Abd. IV length as 6.43–7.50 times as dorsal axial length of Abd. III. Abd. I with 11 (sometimes 12) (a1a, a1–3, m2i, m2–4, m4i, m4p and a5, an unnamed mac sometimes present) mac. Abd. II with six (a2, a3, m3, m3e, m3ea, m3ep) central, one (m5) lateral mac. Abd. III with two (a2, m3) central, four (am6, pm6, m7a, p6) lateral mac (Fig. 77). Abd. IV with two (as, ps) normal sens, 9–12 anterior, 5–6 (A5–6, B5–6, Ae7, A4 sometimes present) posterior and 19–23 lateral mac (Fig. 78). Abd. V with three sens (Fig. 79). Anterior face of ventral tube with 44–55 ciliate chaetae on each side, line connecting proximal (Pr) and external-distal (Ed) mac oblique to median furrow (Fig. 80); posterior face with 5–9 smooth and numerous ciliate chaetae (Fig. 81); lateral flap with 9–11 smooth and 16–25 ciliate chaetae (Fig. 82). Manubrial plate dorsally with 12–17 ciliate mac and 2–4 pseudopores (Fig. 83); ventrally with 39–60 ciliate chaetae on each side (Fig. 84). Dens with 37–66 smooth inner spines (Fig. 85). Mucro bidentate with subapical tooth larger than apical one; tip of basal spine reaching apex of subapical tooth; distal smooth section of dens almost equal to mucro in length (Fig. 86).

##### Etymology.

Named after its locality: Huaping Natural Reserve, Guangxi Guangxi Zhuang Autonomous Region.

##### Ecology.

Found in the leaf litter.

##### Remarks.

The new species is very similar to *H.guangxiensis* sp. nov. and *H.acutus* Jing & Ma, 2022 in the colour pattern, smooth post-labial chaetae, inner teeth on unguis and central chaetae on Abd. IV posteriorly, but can be separated from them by the tenent hair, central chaetae on Abd. IV anteriorly and smooth chaetae on the posterior face of the ventral tube. The detailed character comparisons are listed in Tables 3, 4.

#### ﻿ Homidia oligoseta sp. nov.

Taxon classificationAnimaliaCollembolaEntomobryidae

0EA6870D-D16E-5F7D-A80F-B57A98C52F0A

https://zoobank.org/F75FD2C9-613D-415C-A090-F9BAC8ED6ECB

Figs 87–89 , 90−96 , 97, 98 , 99−106 , 107 , 108 , 109–116 , Tables 3 , 4

##### Type material.

***Holotype*** • ♀ on slide, China, Guangxi Zhuang Autonomous Region, Guilin City, Longsheng Autonomous County, Huaping Natural Reserve, Tianping Mountain, 31-V-2023, 25°37′52″N, 109°54′47″E, 935.4 m asl, sample number 1281. ***Paratypes*** • 2♀ on slides, same data as holotype • 4♀ on slides, China, Guangxi Zhuang Autonomous Region, Guilin City, Longsheng Autonomous County, Huaping Natural Reserve, Tianping Mountain, 1-VI-2023, 25°38′01″N, 109°54′30″E, 707.8 m asl, sample number 1282 • 7♀ on slides, China, Guangxi Zhuang Autonomous Region, Guilin City, Longsheng Autonomous County, Huaping Natural Reserve, Tianping Mountain, Power Station, 2-VI-2023, 25°37′40″N, 109°54′19″E, 682.0 m asl, sample number 1283. All collected by Y-T Ma.

##### Description.

***Size*.** Body length up to 2.21 mm.

**Figures 87–89.**
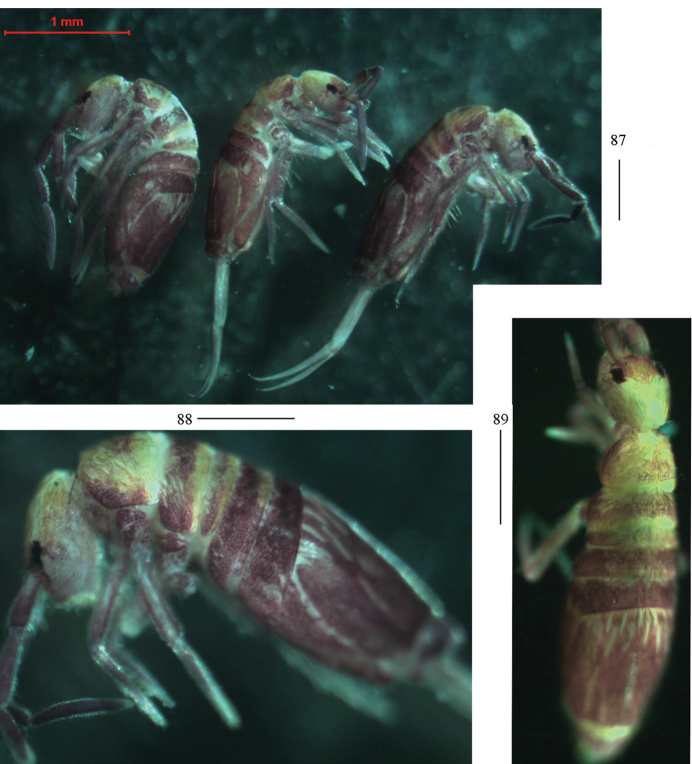
Habitus of *Homidiaoligoseta* sp. nov. (lateral view). Scale bars: 500 μm.

***Coloration*.** Ground colour pale white to yellow; eye patches dark blue; brown to blue-violet pigment present on whole dorsal body, antennae, legs, ventral tube, and manubrium; some unpigmented irregular stripes or spots present on dorsal side of body; Th. II often with less brown pigment (Figs 87–89).

**Figures 90–96.**
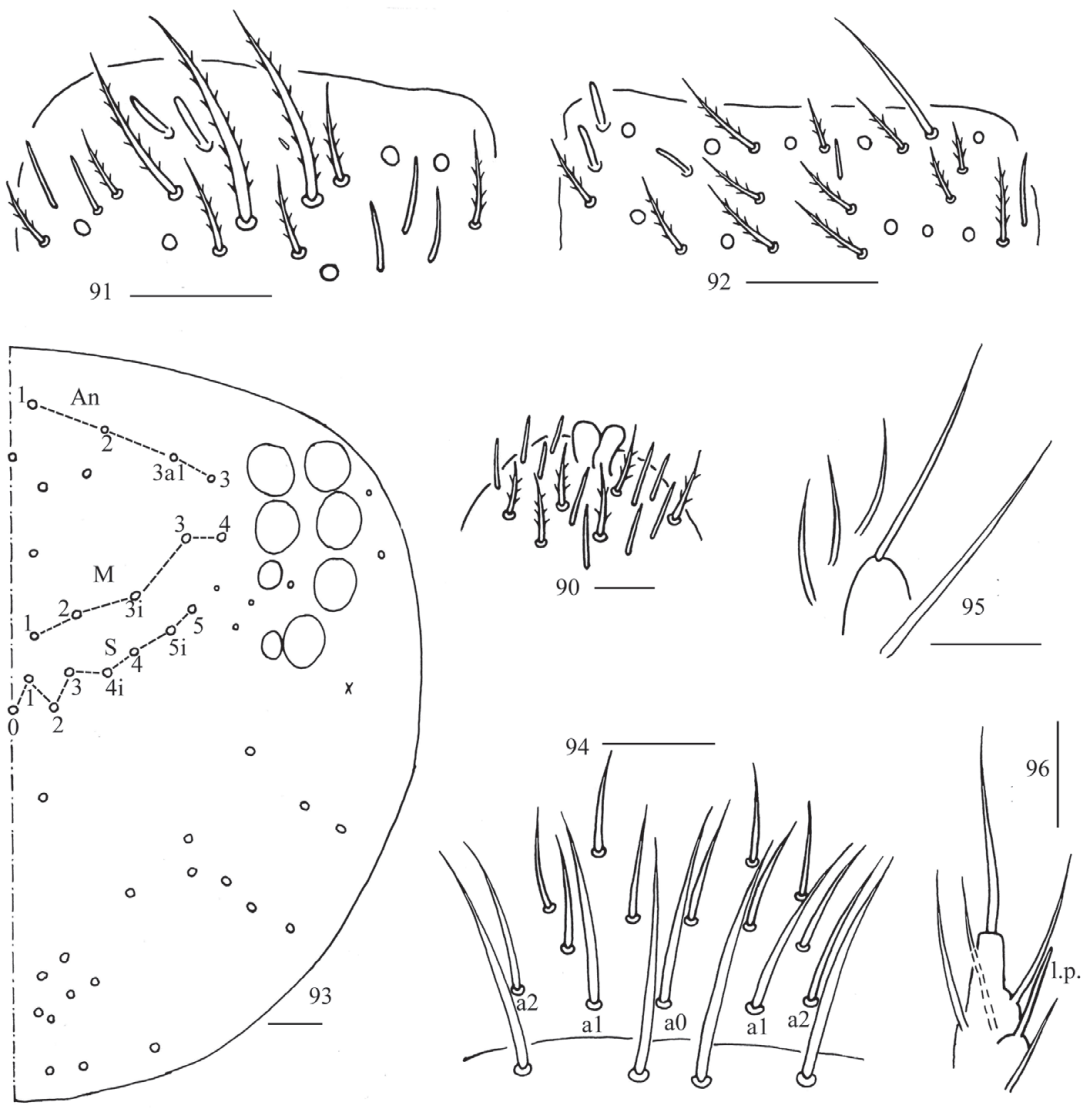
*Homidiaoligoseta* sp. nov. **90** apex of Ant. IV (dorsal view) **91** distal Ant. III (ventral view) **92** distal Ant. II (ventral view) **93** dorsal head (right side) **94** prelabrum and labrum **95** maxillary palp and outer lobe (right side) **96** labial palp. Scale bars: 20 μm.

**Figures 97, 98.**
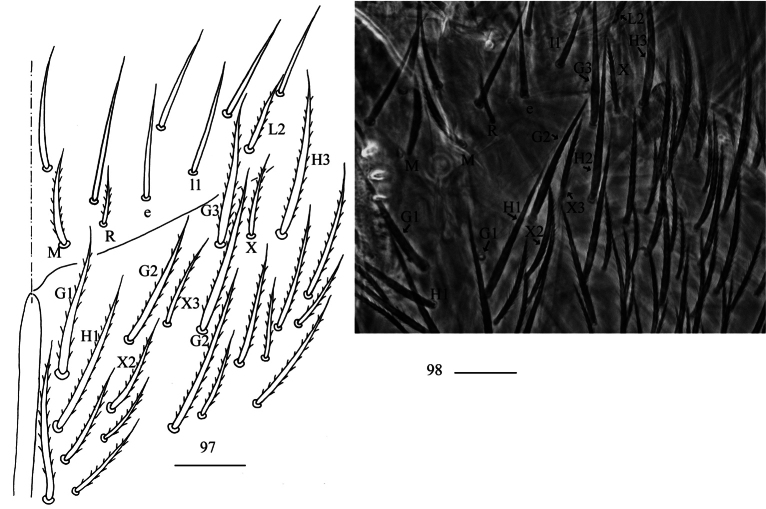
*Homidiaoligoseta* sp. nov. **97** labial and post-labial chaetotaxy (right side) **98** photograph of labial and post-labial chaetotaxy (right side). Scale bars: 20 μm.

***Head*.** Antenna not annulated and 0.56–0.62 times length of body. Ratio of Ant. I–IV as 1.00/1.25–1.47/1.00–1.43/2.00–2.71. Distal part of Ant. IV with many sensory chaetae and normal ciliate chaetae, apical bulb bilobed (Fig. 90). Sensory organ of Ant. III with two rod-like chaetae (Fig. 91). Sensory organ of Ant. II with 2–3 rod-like chaetae (Fig. 92). Eyes 8+8, G and H smaller than others, interocular chaetae as p, r, t mes. Dorsal chaetotaxy of head with four antennal (An), five median (M) and eight sutural (S) mac (Fig. 93). Prelabral and labral chaetae as 4/5, 5, 4, all smooth, a0, a1 longer than a2; labral papillae absent (Fig. 94). Basal chaeta on maxillary outer lobe slightly thicker than as apical one; sublobal plate with three smooth chaetae-like processes (Fig. 95). Lateral process (l. p.) of labial palp E differentiated, as thick as normal chaeta, with tip reaching or exceeding apex of papilla E (Fig. 96). Labial base with MRel_1_L_2_, chaetae e and l_1_ smooth, other ciliate; R 0.53–0.60 length of M. All post-labial chaetae ciliate (Figs 97, 98).

**Figures 99–106.**
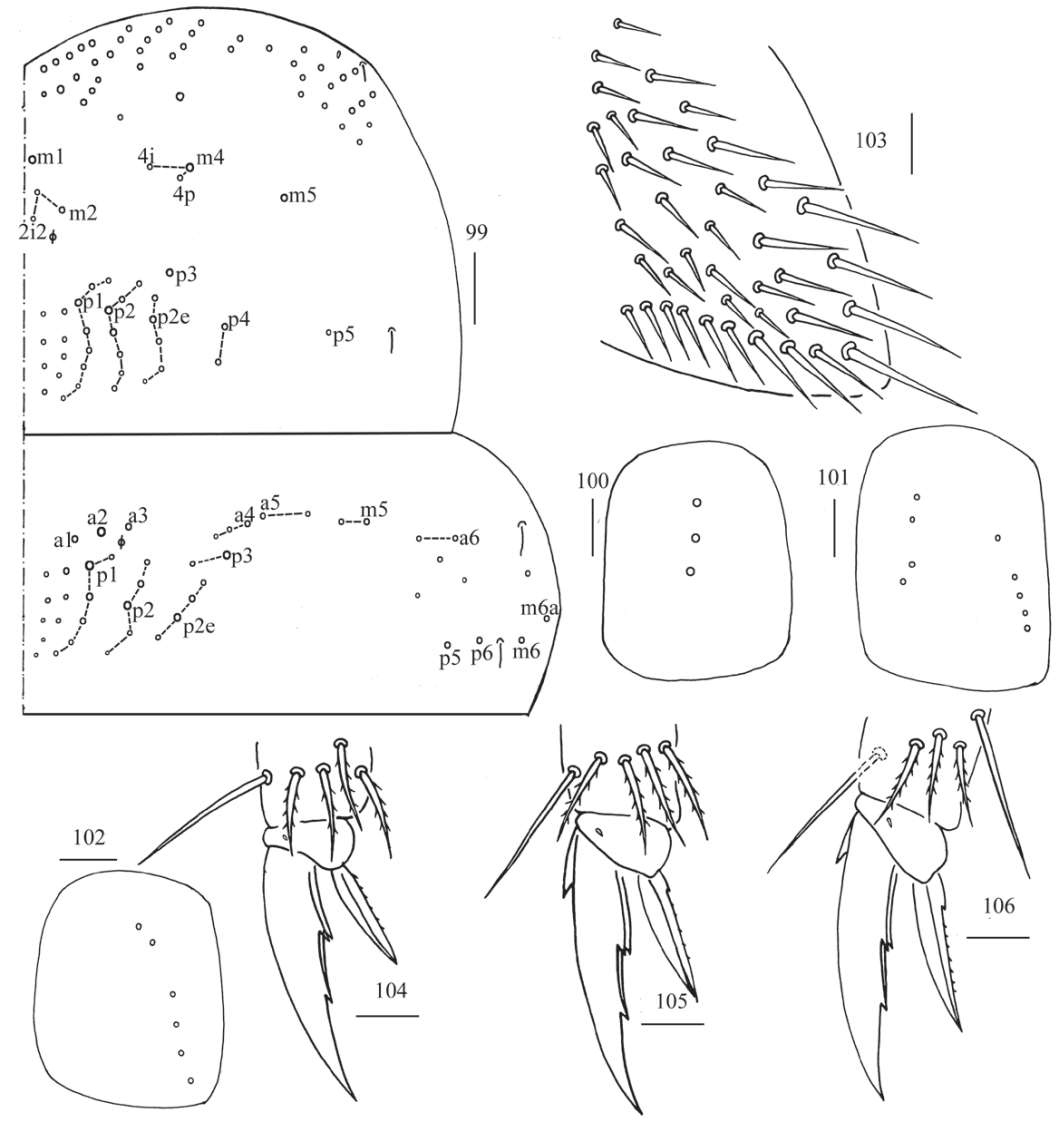
*Homidiaoligoseta* sp. nov. **99** chaetotaxy of Th. II−III (right side) **100, 102** coxal chaetotaxy of fore, middle and hind leg **103** trochanteral organ **104−106** foot complex of fore, middle and hind leg (lateral view). Scale bars: 50 μm (**99**); 20 μm (**100−106**).

**Figure 107.**
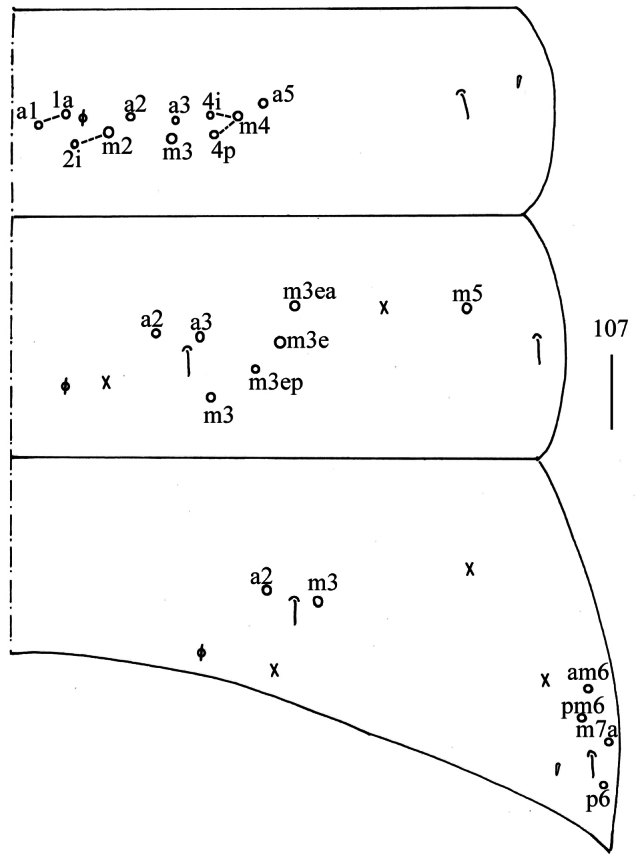
Chaetotaxy of Abd. I−III of *Homidiaoligoseta* sp. nov. (right side) Scale bar: 50 μm.

**Figure 108.**
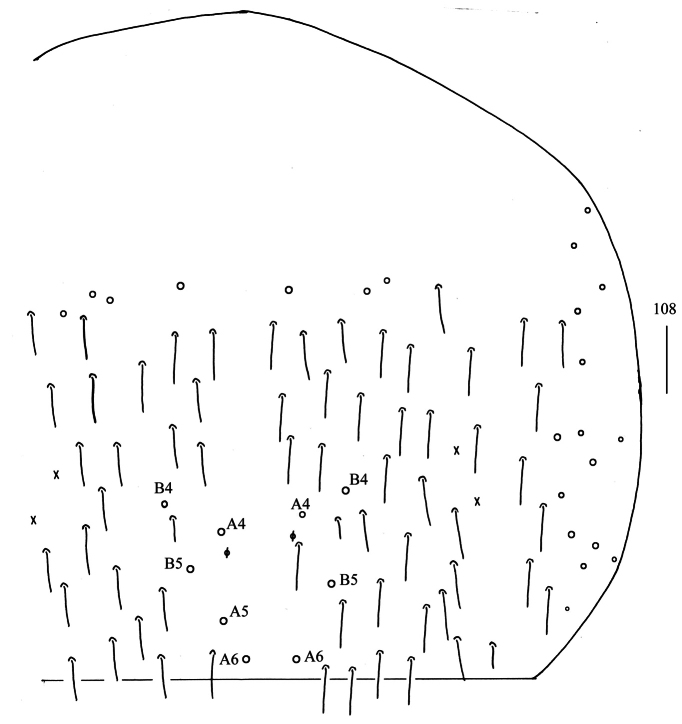
Chaetotaxy of Abd. IV of *Homidiaoligoseta* sp. nov. (right side) Scale bar: 50 μm.

**Figures 109–116.**
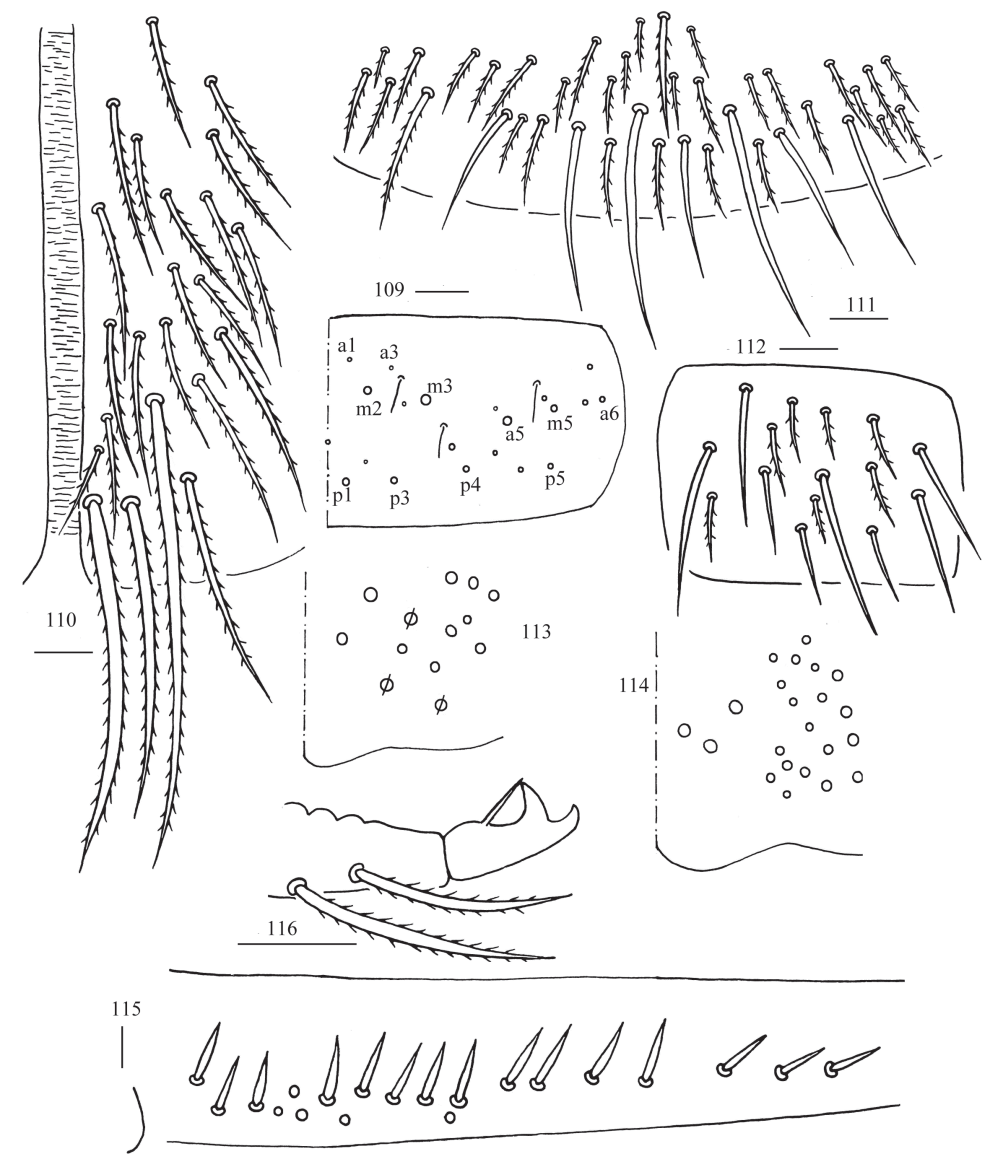
*Homidiaoligoseta* sp. nov. **109** chaetotaxy of Abd. V (right side) **110** anterior face of ventral tube **111** posterior face of ventral tube apically **112** lateral flap of ventral tube **113** manubrial plaque (dorsal view) **114** ventro-apical part of manubrium **115** proximal section of dens (circles also representing spines) **116** mucro Scale bars: 20 μm.

***Thorax*.** Tergal ms formula on Th. II–Abd. V as 1, 0/1, 0, 1, 0, 0, sens as 2, 2/1, 2, 2, 39–55, 3 (Figs 99, 107–109). Th. II with four medio-medial (m1, m2, m2i, m2i2), three medio-sublateral (m4, m4i, m4p), 32–37 posterior mac. Th. III with 45–50 mac (Fig. 99). Coxal macrochaetal formula as 3(4)/4+1, 3(4)/4+2 (Figs 100–102). Trochanteral organ with 40–57 smooth chaetae (Fig. 103). All tenent hairs pointed and 0.60–0.86 length of inner edge of unguis; unguis with three inner teeth, basal pair located at 0.30–0.36 distance from base of inner edge of unguis, unpaired tooth at 0.58–0.71 distance from base; unguiculus lanceolate, outer edge slightly serrate (Figs 104–106).

***Abdomen*.** Range of Abd. IV length as 4.75–6.25 times as dorsal axial length of Abd. III. Abd. I with 11 (10) (a1–3, m2i, m2–4, m4i, m4p and a5, a1a sometimes absent) mac. Abd. II with six (a2, a3, m3, m3e, m3ea, m3ep) central, one (m5) lateral mac. Abd. III with two (a2, m3) central, four (am6, pm6, m7a, p6) lateral mac (Fig. 107). Abd. IV with two (as, ps) normal sens, 3–4(5) anterior, 4(5) (A4–6, B4–5, A5 rarely present) posterior and 11–17 lateral mac (Fig. 108). Abd. V with three sens (Fig. 109). Anterior face of ventral tube with 19–24 ciliate chaetae on each side, line connecting proximal (Pr) and external-distal (Ed) mac oblique to median furrow (Fig. 110); posterior face with 5–7 smooth and numerous ciliate chaetae (Fig. 111); lateral flap with 6–10 smooth and 7–12 ciliate chaetae (Fig. 112). Manubrial plate dorsally with 9–12 ciliate mac and three pseudopores (Fig. 113); ventrally with 22–30 ciliate chaetae on each side (Fig. 114). Dens with 16–31 smooth inner spines (Fig. 115). Mucro bidentate with subapical tooth larger than apical one; tip of basal spine reaching apex of subapical tooth; distal smooth section of dens almost equal to mucro in length (Fig. 116).

##### Etymology.

The name of the new species is derived from the Latin *oligo* and *seta*, which means only a few chaetae are present on each side of the anterior part of Abd. IV.

##### Ecology.

Found in the leaf litter.

##### Remarks.

The new species is very similar to *H.huapingensis* sp. nov. and *H.acutus* Jing & Ma, 2022 in the colour pattern and pointed tenent hair, but can be separated from them by the chaetotaxy of Abd. IV and smooth post-labial chaetae. The detailed character comparisons are listed in Tables 3, 4.

#### ﻿ Homidia acutus

Taxon classificationAnimaliaCollembolaEntomobryidae

Jing & Ma, 2022

176A0036-48FF-5CD1-976B-ED4FD6DEF6E0

Figs 117 , 118–120

##### Material examined.

***Holotype*** and three ***paratypes*** • China, Jiangxi Province, Pingxiang City, Luxi County, Gate of Wugong Mountain, 7-XI-2020, 27°29′27″N, 114°07′33″E, 393.0 m asl, sample number 1229, collected by Y-T Ma.

##### Additions to original description.

**Figure 117.**
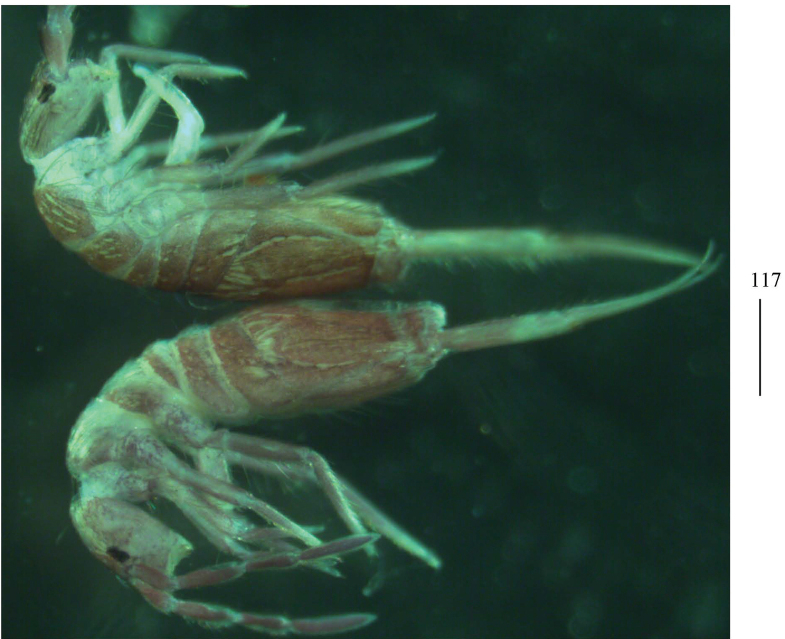
Habitus of *Homidiaacutus* Jing & Ma, 2022 (lateral view). Scale bar: 500 μm.

**Figures 118–120.**
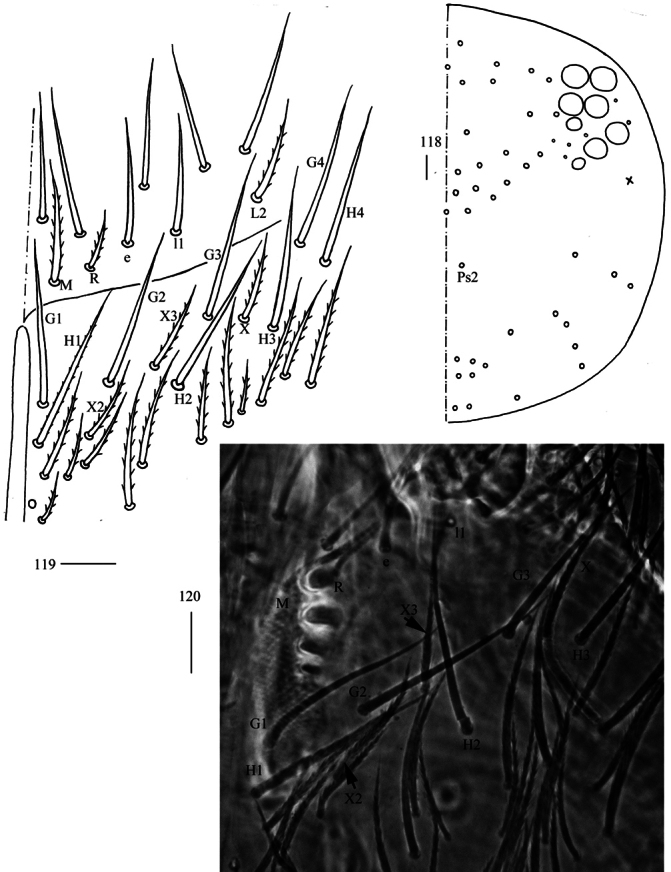
*Homidiaacutus* Jing & Ma, 2022 **118** dorsal head (right side) **119** labial and post-labial chaetotaxy (right side) **120** photograph of labial and post-labial chaetotaxy (right side). Scale bars: 20 μm.

Colour pattern shown in Fig. 117. Ps2 mac present on dorsal head (Fig. 118) (Ps2 is not shown in the original figure because of my carelessness). Post-labial chaetae G_1–4_ and H_2–4_ smooth, sometimes an unnamed chaeta also smooth, chaeta H_1_ slightly ciliate and others normal ciliate (Figs 119, 120).

### ﻿Molecular results

**Figure 121.**
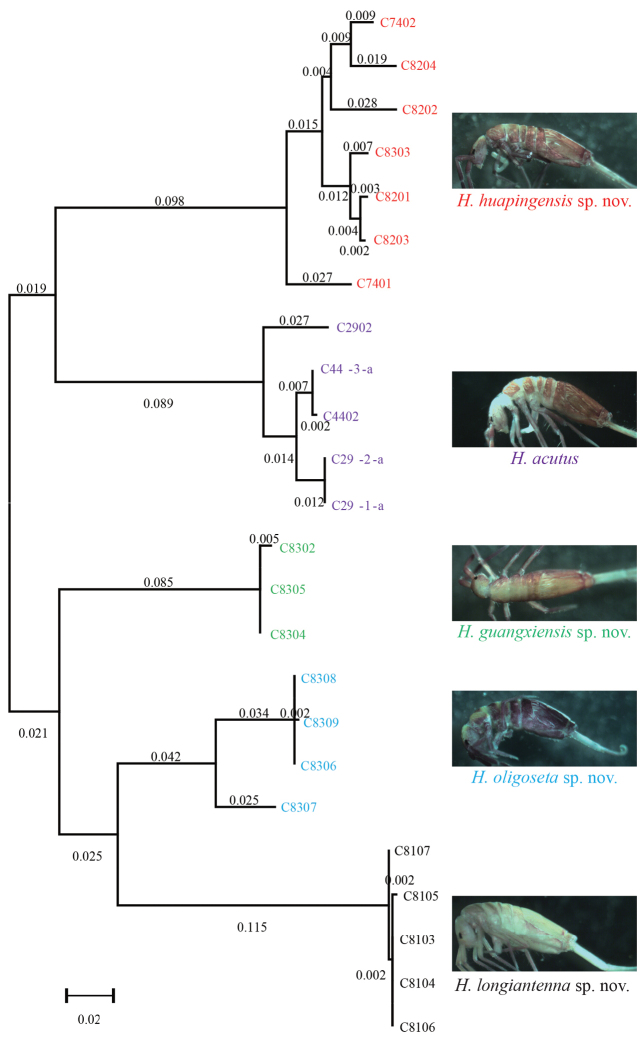
Neighbour joining tree (using K2P model) of five *Homidia* species based on COI sequences.

Sequenced individuals in the present study had a mean K2P distance of COI sequences between 0.177–0.329. The shortest interspecific distance was 0.177 between *H.guangxiensis* sp. nov. and *H.oligoseta* sp. nov. and the greatest was 0.329 between *H.huapingensis* sp. nov. and *H.longiantenna* sp. nov. (Table 5). Therefore, the interspecific distances of COI between the four new species were more than the accepted barcoding gap recently reported for species of Entomobryidae (Zhang et al. 2018) and Tomoceridae (Yu et al. 2018). The molecular distances coincided with the morphological divergences, thus further supporting the separation of the four distinct species (Fig. 121).

**Table 5.** Genetic distances (mean K2-P divergence) within and between species in this study.

Species	*Homidiahuapingensis* sp. nov.	*Homidialongiantenna* sp. nov.	*Homidiaguangxiensis* sp. nov.	*Homidiaoligoseta* sp. nov.	*H.acutus*
*Homidiahuapingensis* sp. nov.	0.005−0.074				
*Homidialongiantenna* sp. nov.	0.305−0.329	0.000−0.004			
*Homidiaguangxiensis* sp. nov.	0.250−0.275	0.225−0.234	0.000−0.005		
*Homidiaoligoseta* sp. nov.	0.257−0.288	0.182−0.197	0.177−0.193	0.000−0.061	
*Homidiaacutus*	0.235−0.261	0.290−0.300	0.235−0.246	0.242−0.259	0.000−0.053

## ﻿Discussion

Like the chaetae on the labial base, the post-labial chaetae are also of various types. Three species (*H.acutus* Jing & Ma, 2022; *H.huapingensis* sp. nov. and *H.oligoseta* sp. nov.) have smooth post-labial chaetae; three species (*H.longiantenna* sp. nov., *H.pseudofascia* Pan, Zhang & Li, 2015 and *H.wanensis* Pan & Ma, 2021) have slightly expanded post-labial chaetae and five species (*H.apigmenta* Shi, Pan & Zhang, 2010, *H.latifolia* Chen & Li, 1999, *H.polyseta* Chen, 1998, *H.qimenensis* Yi & Chen, 1999 and *H.triangulimacula* Pan & Shi, 2015) have strongly expanded post-labial chaetae; most species have normal post-labial ciliate chaetae, or the form of the chaetae is not mentioned.

Colour pattern is a very important character in the taxonomy of Collembola, but some different species may share a very similar colour pattern, such as *H.acutus*, *H.guangxiensis* sp. nov., *H.huangxiensis* sp. nov. and *H.oligoseta* sp. nov. Therefore, it is necessary to combine colour pattern with other characters, such as the tip of the tenent hair, post-labial chaetae and smooth chaetae on the posterior face of the ventral tube, in the taxonomy of Collembola. In addition, COI sequences are useful in separating morphologically similar species.

## Supplementary Material

XML Treatment for Homidia

XML Treatment for Homidia longiantenna

XML Treatment for Homidia guangxiensis

XML Treatment for Homidia huapingensis

XML Treatment for Homidia oligoseta

XML Treatment for Homidia acutus

## ﻿Additional information

### Author ORCIDs

Rong Zhou https://orcid.org/0000-0002-6908-472X

Ling Huang https://orcid.org/0000-0002-6073-9427

Yi-Tong Ma https://orcid.org/0000-0002-8660-0503

### Data availability

All of the data that support the findings of this study are available in the main text.
